# Critical transition and reversion of tumorigenesis

**DOI:** 10.1038/s12276-023-00969-3

**Published:** 2023-04-03

**Authors:** Dongkwan Shin, Kwang-Hyun Cho

**Affiliations:** 1 Department of Bio and Brain Engineering, Korea Advanced Institute of Science and Technology (KAIST), Daejeon, 34141 Republic of Korea; 2 Reasearch Institute, National Cancer Center, Goyang, 10408 Republic of Korea

**Keywords:** Cellular signalling networks, Gene regulatory networks, Targeted therapies, Reprogramming

## Abstract

Cancer is caused by the accumulation of genetic alterations and therefore has been historically considered to be irreversible. Intriguingly, several studies have reported that cancer cells can be reversed to be normal cells under certain circumstances. Despite these experimental observations, conceptual and theoretical frameworks that explain these phenomena and enable their exploration in a systematic way are lacking. In this review, we provide an overview of cancer reversion studies and describe recent advancements in systems biological approaches based on attractor landscape analysis. We suggest that the critical transition in tumorigenesis is an important clue for achieving cancer reversion. During tumorigenesis, a critical transition may occur at a tipping point, where cells undergo abrupt changes and reach a new equilibrium state that is determined by complex intracellular regulatory events. We introduce a conceptual framework based on attractor landscapes through which we can investigate the critical transition in tumorigenesis and induce its reversion by combining intracellular molecular perturbation and extracellular signaling controls. Finally, we present a cancer reversion therapy approach that may be a paradigm-changing alternative to current cancer cell-killing therapies.

## Cancer: Mapping the tipping point to identify reversion therapies

Examining the critical point at which healthy cells become cancerous could provide clues about how to reverse tumor development. Under specific conditions, some cancer cells can revert back to normal, healthy cell types. Dongkwan Shin and Kwang-Hyun Cho at the Korea Advanced Institute of Science and Technology in Daejeon, South Korea, reviewed understanding of cancer reversion, and provide a modeling framework to identify potential reversion therapies. Reversion can occur in leukemia, but reverting cancers with solid tumors remains challenging. ‘Attractor landscape analysis’ maps cells, microenvironments, and signaling networks into a landscape to visualize how cell states change over time. It has been used to examine cancer reversion, but only with data from advanced cancer cells. Conducting this analysis at the tipping point, where a cell undergoes abrupt changes in state, could help identify reversion therapeutic targets.

## Introduction

Cancer is generally caused by genetic alterations that cannot be reversed, such as somatic mutations of oncogenes or tumor-suppressor genes. Therefore, tumorigenesis is considered irreversible. However, cancer cells in normal microenvironments have been shown to revert spontaneously to nonmalignant cells^1^. In 1907, Askanazy reported the phenomenon of tumor reversion: Ovarian teratomas in the embryonic microenvironment evolved spontaneously into differentiated normal cells^2^. Similar findings have been reported in plants, fish, and other organisms, demonstrating that tumor cells can be reprogrammed to acquire a phenotype resembling healthy, normal cells^3^. The experimental and clinical evidence supporting a strategy of reverting cancer cells into normal cells by inducing permanent differentiation has been reported. The most critical experiment supporting cancer cell reversion was performed by Mintz et al., who showed that teratocarcinoma cells injected into blastocysts contributed to normal embryonic development, generating normal organs and tissues^4^. Other attempts to induce the differentiation of acute promyelocytic leukemia (APL) cells, not killing them, were made in the 1970s. On the basis of the results, the use of all-trans retinoic acid (ATRA) with arsenic trioxide (ATO) in treating APL has markedly improved the clinical outcome of APL patients and resulted in cure rates higher than 95%^5–8^. Although differentiation therapy has proven successful in APL, many challenges remain in treating solid tumors with reversion therapy. Most solid tumors are generally characterized by multiple oncogenic signaling pathways and cooperation among these pathways, considerably complicating the effectiveness of differentiation therapy in solid tumors compared to its efficacy against leukemia. Tumor reversion research in the era of molecular biology has focused on the restoration of the normal version of a mutated oncogene or function of a tumor suppressor or the discovery of target molecules that can restore the function of altered phenotypes caused by cancer-driving mutation. Colorectal tumorigenesis can be reversed by restoring the normal function of the aberrantly inactivated tumor suppressor gene *APC*^9^. Telerman et al. described the reversal of malignancy by blocking the expression of specific genes, such as the gene encoding transcriptionally controlled tumor protein (TCTP)^10^. Inhibition of *TCTP* expression reprogrammed p53-mutant leukemia and solid tumor cell lines into “revertant” cells with a suppressed malignant phenotype^11^. Although sporadic phenomenological tumor reversion has been observed in experiments, no systematic approach to understand the mechanisms underlying tumor reversion or to identify molecular targets to restore normal phenotypes to tumor cells has been reported.

A series of studies showing how somatic mutations accumulate during cancer development have important implications for cancer reversion. Martincorena et al. showed that normal cells in sun-exposed skin or esophageal epithelium harbor many cancer-driving mutations but maintain the physiological functions of the epidermis^12,13^. Kaufman et al. demonstrated that, within a cancerized field in which all melanocytes harbored both oncogenic BRAF (V600E) and p53 loss, only a single melanocyte showed a reactivated neural crest progenitor (NCP) state, leading to melanoma initiation via activation of superenhancers at NCP genes^14^. These findings implied that cancer may arise when the accumulation of genetic alterations is accompanied by additional network rewiring, such as epigenetic alterations. It also implies that cells carrying cancer-causing mutations can be reprogrammed to a normal-like state via network modification. Thus, we propose that reversion can occur if complementary network rewiring can be induced in cancer cells.

Many cellular functions are governed by a genome-wide regulatory network that consists of tens of thousands of genes. Furthermore, the behaviors of cancer cells are not regulated by a linear combination of critical genetic alterations but by profound nonlinear cross-regulation between signaling pathways that have been dysregulated because of genetic alterations. In principle, normal phenotypes can be restored by activating bypass or complementary signaling pathways of the complex molecular interaction network to circumvent networks impaired by cancer-causing mutations. In this review, we explore the underlying principle hidden in irreversible transition during the development of cancer and propose a novel theoretical framework for driving cancer reversion based on pharmacological manipulation of the molecular regulatory network involved in cancer and the corresponding attractor landscape. By applying this framework to single-cell data, we propose a system-level approach to identify the molecular candidates that regulate cancer reversion and through which dysregulated cell signaling pathways can be rewired and the hallmarks of cancer can be redressed to re-establish normal phenotypes.

## Cancer reversion: reverting to a normal phenotype

**Fig. 1:**
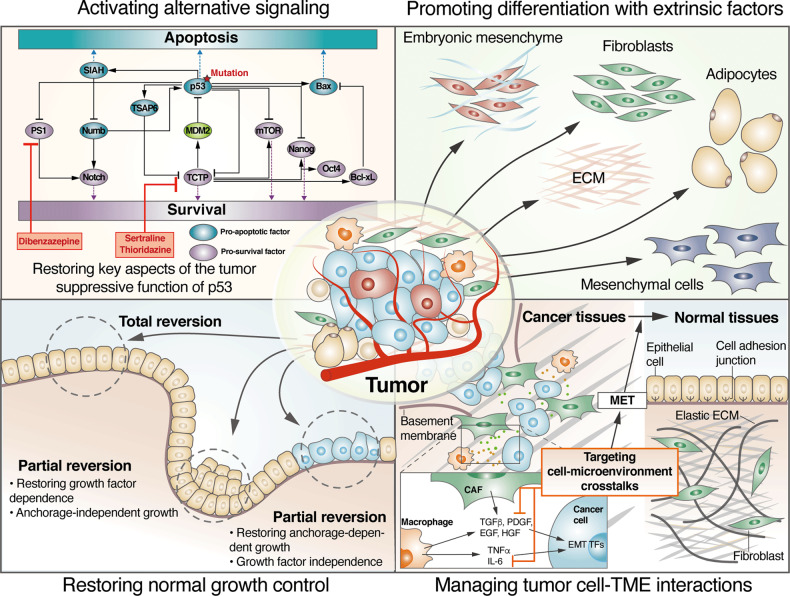
Several perspectives on cancer reversion. The left side shows two methods for manipulating cancer cells to achieve reversion to a nonmalignant phenotype. The right side shows two methods for using external mechanisms to revert cells to a nonmalignant state.

The process of cancer reversion, in a broad sense, involves a cellular reprogramming mechanism by which cancer cells lose their malignant properties and acquire the phenotypic characteristics of normal cells, resulting in the suppression of malignancy. Several perspectives on cancer reversion have been proposed, and they differ primarily with respect to the normal cellular phenotype that has been emphasized (Fig. 1). Telerman et al. introduced three theoretical models of cancer cell reversion^10^: (i) a single event model, in which restoration of a key event involved in the original transformation induces tumor reversion; (ii) a bypass model, in which multiple events target alternative signaling pathways outside of the original transforming pathway for tumor reversion; (iii) a comprehensive model, in which tumor reversion drives cancer cells to transition into a new non-malignant state that is different from the original normal state. For example, malignant cells have been reprogrammed into a new state via the inhibition of TCTP or the overexpression of SIAH-1, which are downstream of inactivated p53^11^. Recently, Lee et al. showed the reprogramming colorectal cancer cells into differentiated normal-like cells by depleting a key regulator, set domain bifurcated 1 (SETDB1), which restored the function of five master regulators that reactivate normal tissue-specific gene expression programs^15^. In contrast, to the manipulation of genes expression in cancer cells, the plasticity of cancer cells can be leveraged to stimulate reversion or reprogramming through extrinsic factors^16^. Examples of external factors that have reprogrammed cancer cells include the embryonic mesenchyme, the extracellular matrix (ECM), and fibroblasts from normal adult tissue and signaling by adipocytes or mesenchymal cells^16^. In some cases, extrinsic signals promote a differentiated state with less malignant properties. However, cancer cell plasticity increases the possibility that a nonmalignant revertant cell can be reversed to a malignant state. On the basis of this concept, Pollack insisted that the most crucial feature of true cancer reversion to a nonmalignant is stability and that stable reversion strategies are superior to strategies that induce differentiation^17^. Partial cancer reversion is an outcome when revertant cells retain certain malignant characteristics. For example, some revertant cells may show suppressed proliferation but continue to exhibit anchorage-independent growth^18^. Complete cancer reversion occurs only when revertant cells exhibit both growth factor dependency and anchorage-dependent growth.

Tumor initiation and progression depend on interactions between a prospective cancer cell and its microenvironment, as well as somatic mutations in the prospective cancer cell. Cell–microenvironment interactions play critical roles in cell fate change by, for example, inducing cellular reprogramming, tumor initiation, cancer metastasis, and cancer reversion^19–21^. Although adult somatic cells can be efficiently reprogrammed into induced pluripotent stem cells (iPSCs) and then redifferentiated to acquire a specific phenotype^22^, in iPSCs placed in the ‘wrong’ environment, reprogramming is terminated, leading to tumor development in various tissues^23^. In contrast, cancer cells seeded in the presence of their normal counterparts^24^ or placed in normal tissues^25^ can be reprogrammed to acquire a normal phenotype.

Cancer cell plasticity is exemplified by the state transitions that enable the metastasis of solid tumors. Many solid tumors arise in epithelial tissues. To become malignant, epithelial cancer cells undergo a transition to a mesenchymal state in a process referred to as the epithelial-to-mesenchymal transition (EMT). Upon reaching a secondary site, cells undergo the opposite transition, the mesenchymal-to-epithelial transition (MET). Bizzarri et al. stressed the relevance of cell–microenvironment interplay in cancer reversion and suggested that the phenotype of a cancer cell can be reversed by properly modifying this interplay to induce the MET by inhibiting the expression of transcription factors related to the EMT^19–21^. Thus, it is important not only to restore the phenotype of cancer cells to that of normal cells but also to manage the extracellular environment to ensure that revertant cells maintain a normal phenotype.

**Fig. 2:**
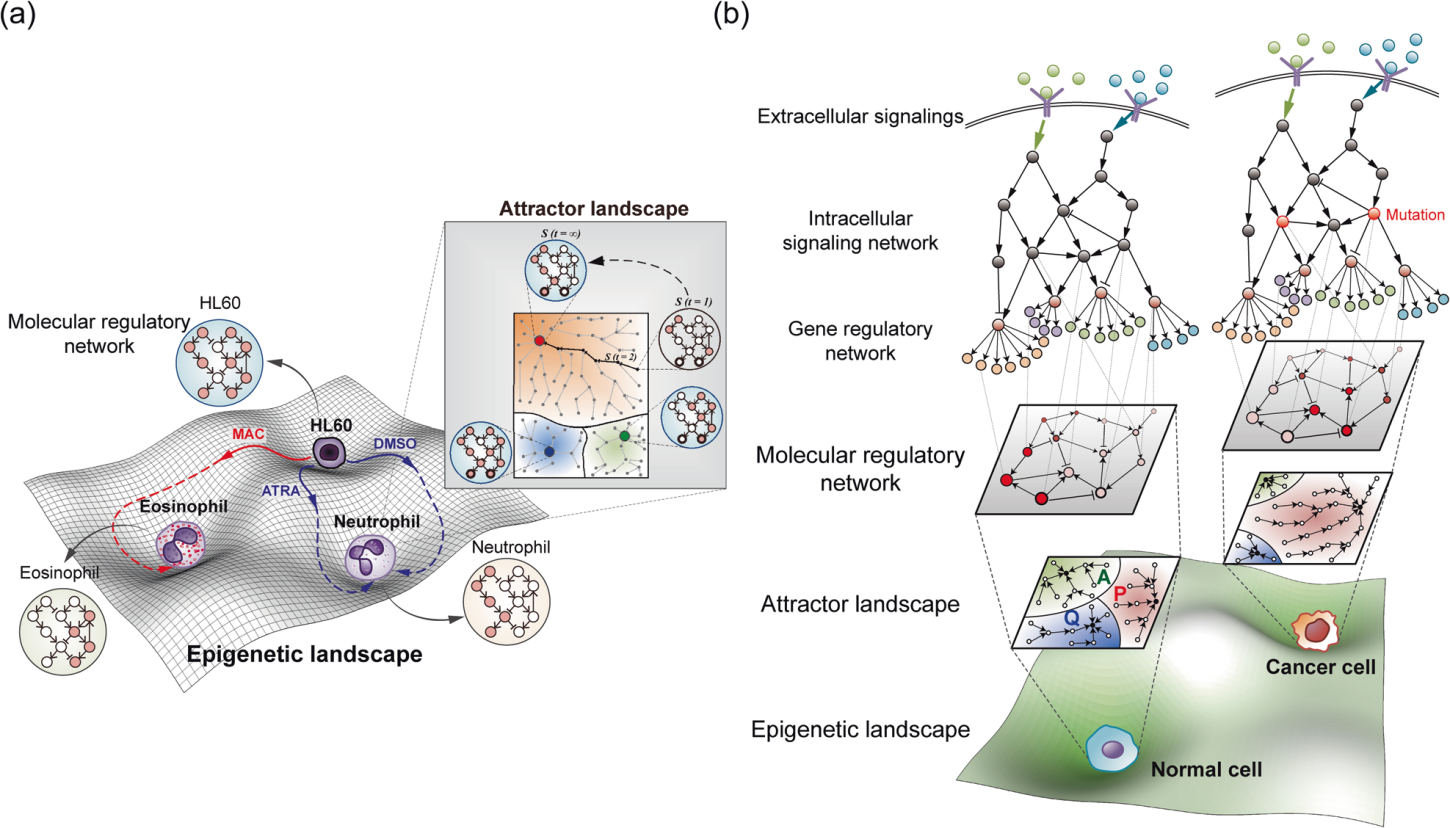
Attractor landscapes of normal and cancer cells. **a** Representation of attractor states within the epigenetic landscape of hematopoietic cells undergoing differentiation. **b** The phenotypic properties of cells can be viewed as attractor landscapes of molecular regulatory networks, including signaling pathways and gene regulatory networks. Cancer cells and normal cells exhibit different attractor landscapes due to network rewiring mediated by somatic mutations, such as the increase in the size of the proliferation-related basin of attraction. Therefore, normal and cancer cells are represented as distinct valleys in an epigenetic landscape. A apoptosis, P proliferation, Q quiescence.

The first step in systemic cancer reversion is the identification of the state of normal or cancer cells and understanding the transitions between them. Although cell states are often characterized by measuring the levels of a small number of key marker genes that are highly correlated with cellular functions, recent high-throughput technologies such as single-cell sequencing have enabled the discovery of characteristic molecules to be greatly expanded^26^. Cells respond to various environmental stimuli through a molecular regulatory network consisting of signaling proteins, transcription factors, and genes. Therefore, the state of a cell at time *t* may be represented by the activities of thousands of molecules, for example, N genes, at time *t*, $x\left( t \right) = (x_1\left( t \right),x_2\left( t \right), \ldots ,x_N\left( t \right))$, and corresponds to a point in an N-dimensional gene expression state space. Therefore, a network state is determined by the underlying gene regulatory network. As a consequence of gene regulatory interactions, a cell state evolves over time with changes in gene expression in a nonlinear time-varying function of all the genes in the network, $\dot x\left( t \right) = F(x_1\left( t \right),x_2\left( t \right), \ldots ,x_N\left( t \right))$, toward a particular convergence state (or a set of states) in the state space, which is called the attractor state (see ref. ^27^ for a comprehensive review on the attractor landscape analysis of gene regulatory networks). Huang et al. experimentally demonstrated that a stable attractor state of a gene regulatory network corresponds to a unique cell phenotype; specifically, the differentiation of human promyelocytic HL60 cells converged to a common neutrophil-like state that was reached via different trajectories in response to different stimuli^28^ (Fig. 2a). Waddington’s epigenetic landscape^29^, a simple and elegant metaphor explaining cell differentiation, is useful for understanding transitions between distinct cell states in a high-dimensional state space. In this metaphor, a particular cell type corresponds to each valley in a hilly epigenetic landscape, and therefore, the cells located in different valleys represent different phenotypic distribution patterns across the surface of the so-called attractor landscape, where each attractor represents a distinct cell phenotype and where the basin (i.e., converging area) of an attractor denotes the probability of convergence on a particular cell phenotype (Fig. 2a).

The abnormal phenotypes of cancer cells, such as uncontrolled proliferation and stimulated angiogenesis, can be represented by attractors in the epigenetic landscape^30^. Because each network architecture is uniquely mapped to one attractor landscape, somatic mutations or drug perturbations induce changes in the attractor landscape. Tracing and controlling these changes enable us to gain a deeper understanding of the development of cancer and identify strategies to reverse cancer cells into cells with a normal state. Notably, there have been many attempts to apply attractor landscape analysis to cancer therapy^3,31–36^. The attractor analysis of p53 network dynamics suggested that combined inhibition of specific components in the network enhance the apoptotic response to DNA damage^31,36^. Recent efforts have been directed to a control-based reversal of irreversible biological processes, including cancer, by manipulating the attractor landscapes of cellular networks^32–35^. The simulation analysis of a large-scale Boolean network model of colorectal cancer showed that the identified molecular targets of the reverse control were highly enriched in approved anticancer drug targets^33,34^. In a recent network analysis of breast cancer, potential targets for reprogramming basal-like breast cancer cells into luminal A subtype cells were identified and validated by experiments^32^. These efforts to find cancer reversion targets based on attractor landscape analysis have led to insights that indicate differences between cancer cell reversion and current cancer therapies aimed only at killing cancer cells. Nevertheless, these approaches are fundamentally limited since the analyses were mostly focused on cancer cells without fully analyzing the state of the normal cells, which are the cells obtained after the application of the cancer reversion strategy. Therefore, previous efforts to identify targets for cancer reversion by changing the shape of the attractor landscape of cancer, for instance, by increasing the basin of the apoptosis attractor and decreasing the basin of the proliferation attractor, likely resulted in the nonoptimal identification of either anticancer targets or reversion targets. Next, we introduce the critical transition in tumorigenesis and suggest new ways to overcome the aforementioned limitations of previous studies based on attractor analysis.

## Critical transition in tumorigenesis

**Fig. 3:**
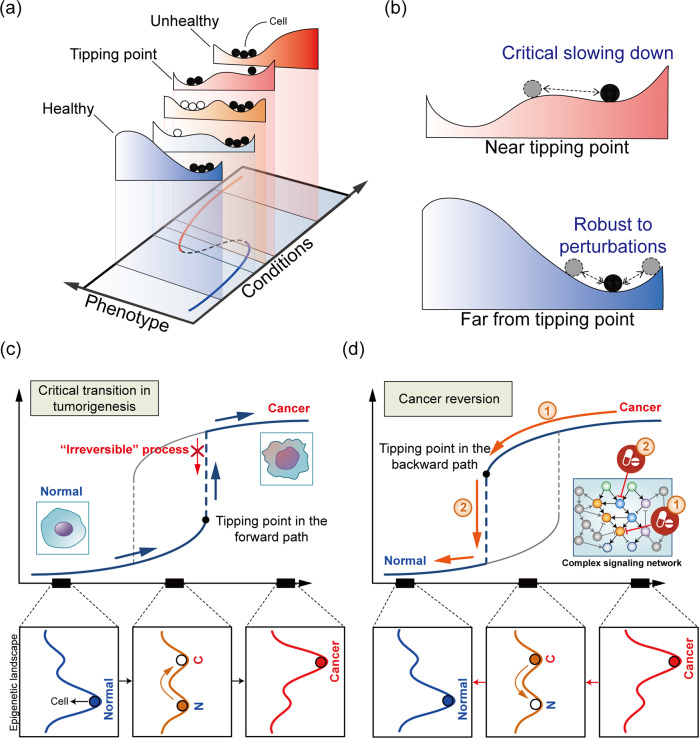
Critical transition in tumorigenesis and cancer reversion. **a** Epigenetic landscape and critical transition. The state of a dynamic system is represented by the position of a ball on a quasipotential landscape, with valleys corresponding to the basins of attraction of the stable states in the system. Changing the condition via environmental perturbation leads to the modification of the landscape, changing it from one with healthy to one with unhealthy phenotypes. Solid circles represent cells occupying the attractor state, whereas blank circles represent cells that are likely to occupy the state induced by certain perturbations or stochastic noise. **b** The response of a cell to perturbations near the tipping point (top) or far from the tipping point (bottom). **c**, **d** Critical transition in tumorigenesis (**c**) and two-step strategy for cancer reversion (**d**). Changes in the epigenetic landscape are illustrated along the paths. N normal, C cancer.

During the transformation of a normal cell to a cancer cell through somatic mutations, signaling pathways and gene regulatory networks are altered. This network rewiring drives cancer cells to exhibit abnormal or malignant phenotypic characteristics. When evaluated in the context of an epigenetic landscape, cancer cells, and normal cells exhibit completely different attractor landscape patterns (Fig. 2b). An attractor state (a stable cell state) typically requires feedback loops to function in the underlying molecular regulatory network^37,38^. For example, positive feedback loops can generate multistability, resulting in different phenotype states^39–41^. Complex biological networks composed of abundant interconnected feedback loops often cause multistability, as well as inherent nonlinearity and functional redundancy. These features contribute to cell homeostasis, enabling a cell to consistently return to a stable state in response to external changes. However, these homeostatic mechanisms can induce a sudden change in the cell state in response to gradual changes in external conditions, such that at a certain tipping point, a cell enters a different stable state (Fig. 3a). Therefore, switching between two stable cell states, such as between normal and cancer cell states, may represent a critical transition from one attractor to another attractor in the epigenetic landscape^42^.

A critical transition is a concept established in physics and chemistry and is observed mainly in ecosystems, climates, and social systems. A feature of a critical transition is a “critical slowing down” phenomenon, in which the recovery of the system to the original stable state after perturbations is slowed because the original state becomes increasingly unstable as the system approaches the critical point (Fig. 3b). Therefore, near the tipping point, the system exhibits alternative stable states, meaning that the system can function in more than one stable state under the same external condition. Using data from single-cell analysis, the concept of a critical transition or tipping point has been applied to complex biological processes, such as differentiation or tumorigenesis^43^. For example, during the commitment of blood progenitor cells to an erythroid or myeloid lineage, cells undergo a critical state transition preceded by the destabilization of their attractor state in the high-dimensional state space^44^. Mojtahedi et al. introduced a new quantitative index to detect critical transitions in a high-dimensional state space. The index is based on important features identifiable at the tipping point, such as a reduction in cell‒cell correlation and an increase in gene‒gene correlation^44^. A single-cell proteome study in cells undergoing carcinogen-induced tumorigenesis suggested a critical transition during tumorigenesis^45^, with Poovathingal et al. observing phase coexistence and divergent correlation lengths, which are statistical indicators of a critical transition. Although a critical transition in a biological process may imply that tumorigenesis is irreversible and that cells cannot return to the original state on the tumor evolutionary trajectory, the critical transition point may provide clues into the mechanisms by which the original phenotype can be restored^35^. At the tipping point, initial and final states can coexist. Hence, knowledge of the tipping point during cancer development can provide critical information for cancer reversion to a normal cell state.

Conventional anticancer treatment studies have been conducted with advanced tumor cells. However, these cells do not provide sufficient information on the precancerous state of the cells or the mechanisms underlying the transition to the cancer cell state. Without this information, researchers can only block proliferation or induce cell death to control cancer progression or regression. By identifying and understanding the tipping point of a critical transition, we can conceptually explore different strategies to control cancer progression or regression through cancer reversion.

## Cancer reversion based on the critical transition in tumorigenesis

Cancer is an extremely complex disease that exhibits a high level of protection against external perturbations. This protection from perturbation enables cancer cells to proliferate and survive despite treatment. Using the attractor landscape paradigm, cancer cells are represented by cells in a stable attractor state of a complex dynamic system. Therefore, the transition from the attractor state of a normal cell to the attractor state of a cancer cell represents a critical transition (Fig. 3c). Viewing tumorigenesis as a critical transition may provide new insight into the development of different kinds of drugs and therapies based on cancer reversion. One of the characteristics of a critical transition is that when approaching a tipping point, the system is extremely sensitive to miniscule changes in environmental conditions. Cancer cells harbor many somatic mutations, but only a few critical “driver” mutations contribute to cancer formation; the others are considered “passenger” mutations^46,47^. Driver mutations confer a selective advantage to a cancer cell by inducing a dramatic change that affects the entire molecular interaction network^48^. After entering a robust cell state through a critical transition, cancer cells maintain the acquired state even after the withdrawal of the driving sources that induced tumor formation because of a high barrier in the potential energy between normal and cancer cell states. Therefore, for a cell to return to its original stable (normal) state after it has acquired a stable cancer state phenotype, sufficient external changes or stimuli are required to push the system across the transition threshold (a tipping point along the backward path) toward the original state (Fig. 3d). In other words, the network rewiring used to induce the backward transition from the cancer states needs to overwhelm the effects of the driver mutations that caused tumorigenesis.

Because cell in two distinct states can coexist around a tipping point during a critical transition, we propose a two-step strategy for inducing cancer reversion. The first step is to drive cancer cells back to the bistable state near the tipping point by targeting molecules in intracellular molecular networks that can reshape the attractor landscape of the cancer state. In the attractor landscape, this reshaping involves a decrease in the basin size of proliferative attractors and an increase in the basin size of apoptotic attractors. The state of a cell in a bistable state can be changed via stochastic fluctuations that cause the cell to jump to an adjacent state without requiring network parameter changes because of the low potential energy barrier. Thus, cells can stochastically fluctuate between normal and cancer states^43^. The second step is to drive the bistable state in a single direction—allowing only the cancer-to-normal transition while blocking the normal-to-cancer transition.

**Fig. 4:**
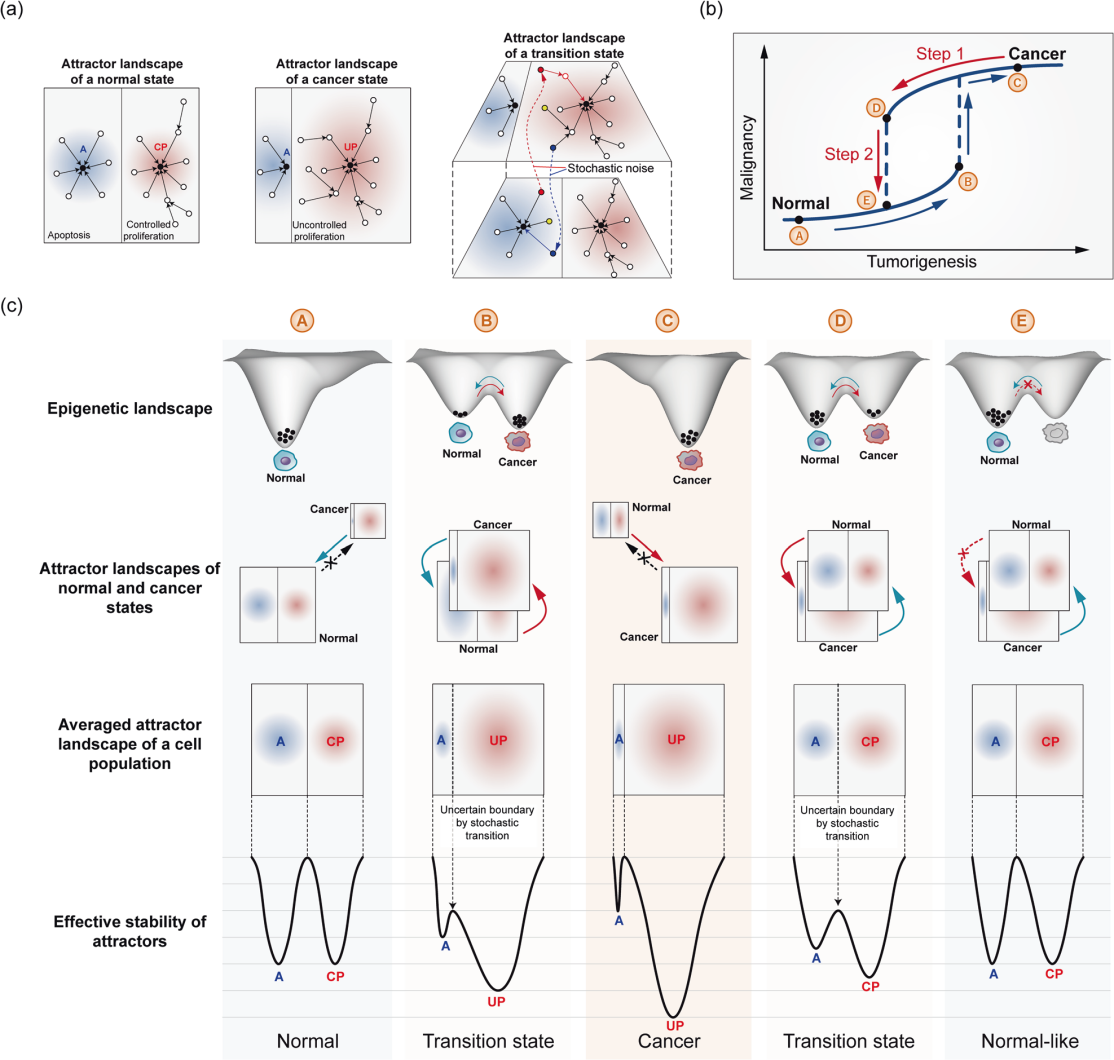
Attractor landscapes of normal and cancer cells during tumorigenesis and cancer reversion. **a** Attractor landscapes of normal, cancer, and transition states. Circles represent network states of a cell. In a transition state, a network state of an attractor landscape can jump to the same network state in an adjacent landscape because of stochastic noise; therefore, the network state can converge to a completely different attractor state. Attractor states: A apoptosis, CP controlled proliferation, UC uncontrolled proliferation. **b** Distinct forward and backward paths along the transition trajectories from the normal to the cancer states and the reversion trajectories from the cancer to the normal states. **c** Epigenetic landscapes, attractor landscapes, and effective stability of the attractors during tumorigenesis and cancer reversion. Points A to E correspond to positions along the graph shown in (**b**). Each valley in the epigenetic landscape represents a cell state, either a normal or cancer state, where cells designated as black dots can either maintain a stable state or stochastically transition between stable states during a transition. The average attractor landscape of a cell population in each condition can be obtained through the linear combination of normal and cancer attractor landscapes, where a solid line between the A and CP or UP attractor states indicates a high barrier between states and a dashed line indicates a barrier low enough for cells to move between attractor states.

The basis of a two-step process for cancer reversion can be explained by tracing changes in the epigenetic landscape, the attractor landscape of normal and cancer cells, and the stability of cancer-related attractors during the cancer initiation process and the reverse process (Fig. 4). A well-balanced distribution of phenotypes, such as proliferation and cell death, is maintained in the attractor landscape of normal cells, whereas in cancer cells, the basin of attraction associated with a cancer hallmark, such as uncontrolled proliferation, may be a large proportion of the attractor landscape. The attractor landscape of a transition state, where normal and cancer cell states may coexist, can be viewed as a conditional framework in which two landscapes, those of the normal and cancer cell states, are in such close proximity that cells can undergo a stochastic transition between these two landscapes (Fig. 4a). Therefore, a snapshot of a transition state after the first reversion step may show two types of cells in each valley and these cells may correspond to normal and cancer cell states in the epigenetic landscape. Since this state (D in Fig. 4b) is expected to be closer to the normal state than to the tumorigenesis tipping point (B in Fig. 4b), the attractor landscape at a population level is likely to exhibit a phenotype distribution similar to that of the normal cell state, except that the barrier between the attractors of cell death and proliferation is low, implying that a cell population may develop into cancer cells (see D in Fig. 4c). In addition, the second step, in which the transition from the normal state to the cancer state is blocked, effectively increases the barrier to transition between phenotypes, thereby inhibiting an increase in uncontrolled proliferation and maintaining a normal-like state. The final state is not exactly the same as the original state, and it does not have to be, as long as the new state is in a normal attractor landscape (see E in Fig. 4c). An attractor landscape analysis can reveal the targets of the first and second steps needed to achieve stable cancer reversion.

## Defining targets for two-step cancer reversion

**Fig. 5:**
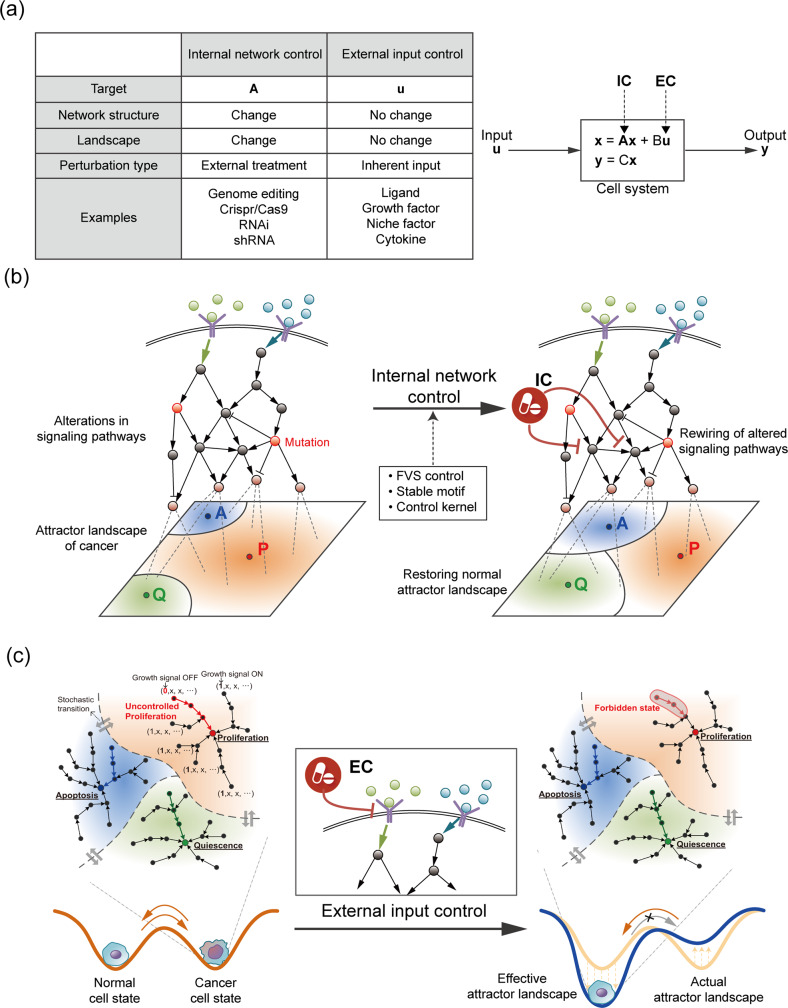
Internal network control and external input control for cancer reversion. **a** Defining control strategies in a cellular system. A cellular system is represented by ${{{\dot{\boldsymbol x}}}} = f({{{\boldsymbol{x}}}},{{{\boldsymbol{u}}}})$, where ***x*** is the n-dimensional state vector of genes and *f*(***x***, ***u***) is a vector field that describes the dynamics of the system related to the input signal ***u***, which is an m-dimensional control vector of genes. Near an equilibrium point in the nonlinear system, the vector field *f* can be approximated by *A****x*** + *B****u***, where *A* is an adjacency matrix and *B* is an appropriate dimensional matrix of constant coefficients of input weights. The system output can be described as a simple weighted combination of the state variables, ***y*** = ***Cx***. **b** An internal network control (IC) mediates alterations in a signaling pathway to restore the attractor landscape of normal cells. **c** External input control (EC) that effectively modifies the epigenetic landscape to make the normal state more stable. An example of EC is a growth factor receptor inhibitor that prevents cells from maintaining a cancer state by completely blocking a specific trajectory related to uncontrolled proliferation.

Cells function through complex interconnected pathways that are regulated by inputs such as signals from tissues or the tumor microenvironment (TME). The outputs of these functions are cell behaviors, such as quiescence, proliferation, and apoptosis. Most cellular systems exhibit nonlinear dynamics. However, as the controllability of nonlinear systems is structurally similar to that of linear systems^49^, cellular systems can be approximately represented by a linear time-invariant (LTI) system: ${{{\dot{\boldsymbol x}}}} = A{{{\boldsymbol{x}}}} + B{{{\boldsymbol{u}}}}$, where ***x*** is an n-dimensional state vector of molecules, *A* is an adjacency matrix of the molecular interaction network, and *B* is a constant coefficient that weights the input stimulus, ***u*** (Fig. 5a). By introducing this linear model, we can highlight the difference between the two steps of cancer reversion on the basis of the mode of control and the targets. To complement this simplicity, a dynamic network model reconstructed on the basis of a case study, which is not a simple linear model but is, in fact, a nonlinear model defined by a Hill-type function, is presented in the following section.

### Step 1: Internal network control (IC)

To destabilize a stable and even drug-resistant state of cancer cells, we propose that the first step involves rewiring the cancer signaling networks to restore the attractor landscape of normal cells. One obvious approach involves reshaping the landscape to re-establish the exact same attractor (regulatory network state) as that of the original normal cells. However, this type of precise control is unrealistic in practice because altered genes cannot be restored to their unaltered forms. Therefore, intracellular pathways cannot be perfectly restored to be identical to the pathways in the cells before genes were altered. Fortunately, we can use an alternative strategy to establish an attractor landscape with a critical attractor basin size similar in proportion to that in the original attractor landscape (Fig. 5b). The critical attractors are hallmarks of cancer, such as proliferation, apoptosis, quiescence, and metabolism. However, the state transition trajectories to this new normal state can be different from the trajectories that led to the cancer state (Fig. 4b). Several complex network control methods have been developed to drive an initial state to a desired attractor state based on feedback vertex sets^50^, control kernels^51^, and stable motifs^52^. By applying these control methods to the intracellular signaling network, cancer cells may lose their cancer hallmark features and gain characteristics of normal cells, thereby entering a transition state where normal and cancer cell states coexist. These control methods involve alterations to the intracellular molecular network (*A*) in the system equation; therefore, we refer to this first step as the internal network control (IC).

IC drives the system into a transition state in which a cancer cell can stochastically switch into a normal cell and vice versa. In living cells, this unstable transition state is populated with cells with heterogeneous transcript and proteome profiles. Experimental support for this transcriptional heterogeneity has been observed in hematopoietic stem cells undergoing state switching during differentiation^44,53^. Experimental support for this proteome heterogeneity and cells coexisting in initial and final states has been reported during the cell transition during chemically induced carcinogenesis^45^. In the transition state, the transition barrier between the normal and cancer states is relatively low. Therefore, the IC is insufficient to achieve stable cancer reversion. A second step is needed.

### Step 2: External input control (EC)

To reduce cell state instability, we need a second control strategy that blocks the transition from the normal cell state to the cancer cell state, effectively making the valley of the normal cell state deeper than that of the cancer state (Fig. 5c). When an attractor landscape has been established by an IC such that the cells acquire a normal phenotype, for example, via the elimination of a persistently activated path to growth factor-independent proliferation, then, the second step involves eliminating the input that induces acquisition a cancer phenotype, which in this example may be eliminating stimuli of growth factor-dependent proliferation. Because this control strategy involves manipulating an input (*u*) to the network, we refer to the second step as external input control (EC). Changes to the input also affect the initial state of intracellular pathways. Therefore, the EC can be used to establish an initial state that enables an IC to induce a stable cell transition from a cancer state to a normal-like state.

### IC and EC candidates

The IC strategy involves the perturbation of (*A)*, resulting in a change in the structure of the molecular regulatory network by targeting specific molecules or their interactions within the network. The altered network structure reshapes the attractor landscape of cancer cells, pushing them toward a transition state. In contrast, the EC strategy targets system input (***u***). Thus, an EC influences the initial value of the system so that the cells converge at a nonmalignant attractor state in the given attractor landscape after IC treatment. An attractor landscape analysis enables the identification of inputs and molecules within the regulatory network that can be targeted by ICs and ECs.

Experimentally, targets of ICs are often validated with methods for modifying gene expression, gene‒gene interactions, or an encoded product; these modifiers include siRNA, shRNA, the CRISPR/Cas9 system, proteolysis targeting chimeras (PROTACs), aptamers, and enzyme inhibitors. ECs are typically validated by exposing the cells to ligands, growth factors, niche factors, or cytokines, which mediate extracellular stimulus-induced signaling that represent inputs into the regulatory network.

**Fig. 6:**
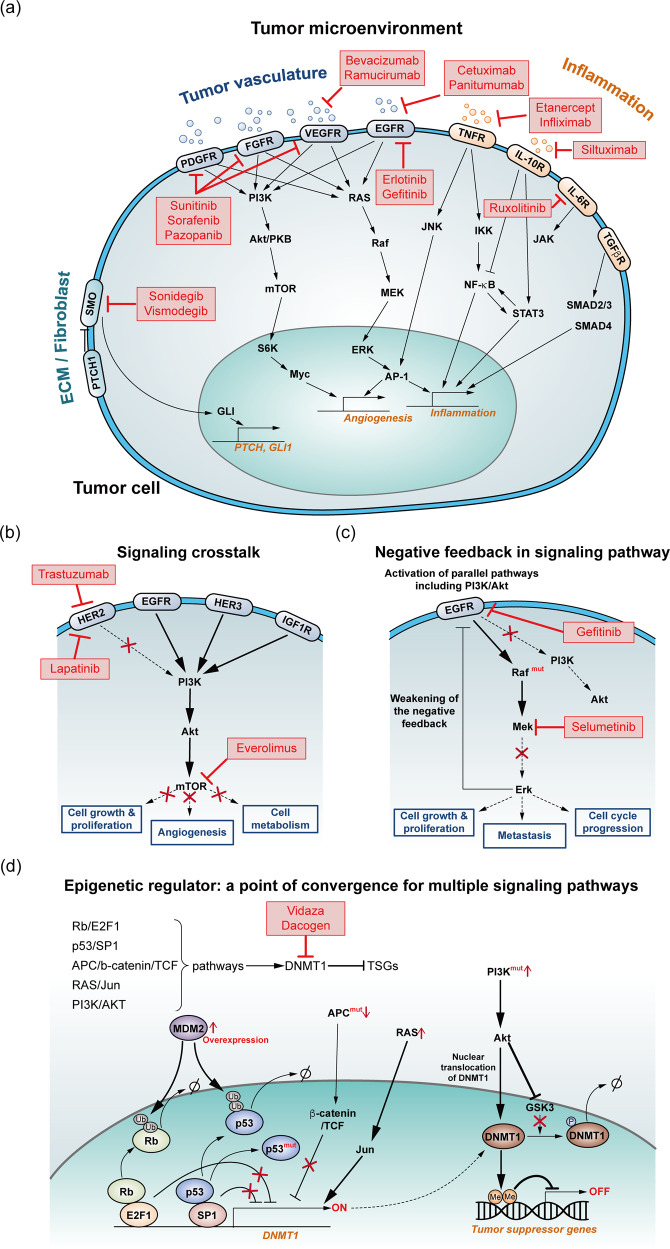
Candidate target and representative ICs, ECs, and IC and EC combinations. **a** FDA-approved drugs as potential candidates for ECs that inhibit crosstalk between tumor cells and the TME; these candidates include tumor vasculature, ECM, and inflammatory factors. **b** Combining an IC and EC to enhance therapeutic responsiveness by blocking signaling crosstalk that converges to a cancer pathway. **c** Combining an IC and EC to overcome negative feedback in the MAPK signaling pathway. **d** Targeting tumor checkpoint modules. The epigenetic modulator DNA methyltransferase 1 (DNMT1) is downstream of multiple cancer-relevant pathways and promotes tumorigenesis. Targeting DNMT1 restores the normal activity downstream of multiple cancer-associated signaling pathways.

Candidate ICs and ECs in cancer reversion include various drugs that are used for cancer regression. Potential EC candidates are therapeutics that interfere with the function of cell surface receptors, such as the receptors that respond to growth factors, cytokines, or molecules in the extracellular matrix (Fig. 6a). These drugs include the FDA-approved agents bevacizumab and cetuximab, which interfere with the interaction between tumor cells and the TME. IC candidates are drugs that interfere with intracellular signaling molecule activity, such as inhibitors of the mitogen-activated protein kinase (MAPK) cascade or inhibitors of the phosphoinositide 3-kinase/AKT/mTOR pathway.

Combining an IC and an EC is a strategy for overcoming therapeutic resistance. For example, the combination of the IC drug everolimus, an mTOR inhibitor, with the EC drug trastuzumab effectively inhibits the downstream PI3K/AKT signaling pathway, blocking other inputs that activate PI3K/AKT signaling^54^ (Fig. 6b). In addition, several EC/IC combination therapies, in addition to trastuzumab and everolimus, such as erlotinib and everolimus, and neratinib and temsirolimus, may prevent alternative signaling events that promote cell proliferation and survival^55^. A combination of MEK and EGFR inhibitors blocks the negative feedback loop to EGFR, providing synergistic benefit^56,57^ (Fig. 6c). The extent to which these combination EC/IC therapies promote both cancer reversion and cancer regression by cell death or immune system-mediated elimination remains unknown. However, the combination strategy is effective in many cancers, including breast cancer, colorectal cancer, and non-small cell lung cancer^58^.

Another strategy for manipulating the effect of an EC is based on leveraging epigenetics. Tumor checkpoint modules represent small, highly connected, and autoregulated sets of proteins that canalize the effects of genomic alterations and other aberrant signals to orchestrate downstream transcriptional programs involved in tumor progression^59,60^. Therefore, it may be difficult to change the phenotypic properties of cancer cells with treatments that only regulate signaling upstream of altered signaling pathways. Adding drugs that target epigenetic regulators to treatments that control signaling pathways or their upstream factors may alter the attractor landscape of the network and may be effective in driving normal-like state acquisition (Fig. 6d). In addition, epigenetic regulators that are part of a tumor checkpoint module are often triggered downstream of multiple signaling pathways. Therefore, controlling a few signaling pathways may not restore the expression of tumor suppressor genes. Drugs such as Vidaza and Dacogen target DNMT1, an epigenetic regulator that is part of a tumor checkpoint module. Vidaza and Dacogen are recognized as effective therapies for several types of cancer and function by reactivating tumor suppressor genes^61^.

The aforementioned and other FDA-approved epigenetic therapeutics, including methylation inhibitors and histone deacetylase inhibitors, may be used with EC/IC therapies for cancer reversion.

## Implementation of a cancer reversion strategy on the basis of single-cell data

**Fig. 7:**
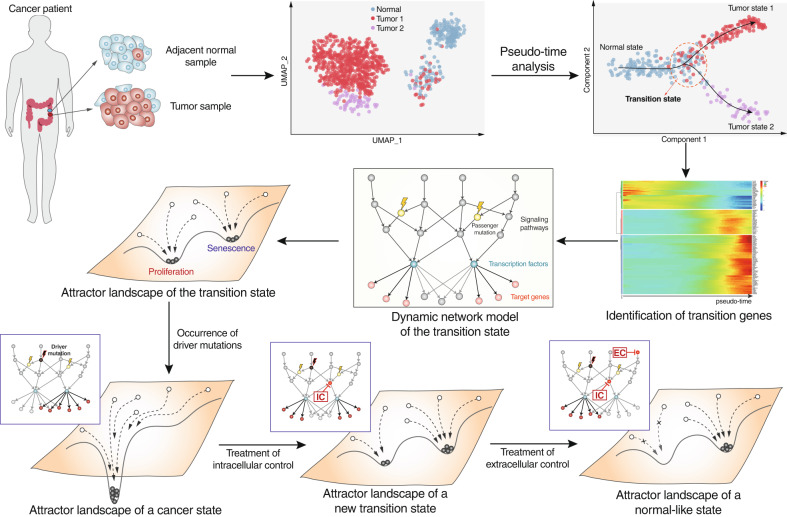
Overview of ways to implement a control strategy for cancer reversion using single-cell genomic data and attractor landscape analysis. Transcriptomes of single cells obtained from tumor and matched normal samples were clustered using dimension reduction techniques, and then cell identity was determined by measuring the expression levels of known cell marker genes. Cell populations, such as normal epithelial and tumor cells, can be ordered along tumorigenic trajectories using pseudotime analysis. The pseudotime information can also be used to construct a dynamic network model of the transition state through the identification of transition-related genes associated with tumorigenesis. Through a dynamic network model, the attractor landscape of a transition state can be identified. The attractor landscape of the cancer state can be determined by adding driver mutations to the network model. The dynamic network model and attractor landscape of cells in a cancer state can help to identify optimal IC and EC targets through the application of one or more control theories.

Applying an IC and EC to mediate cancer cell reversion and confirming such a mechanism of action are challenging. We cannot deduce the ultimate target, that is, a normal cell state, of a cancer reversion strategy on the basis of a cancer sample. Identifying a new transition state after IC treatment without knowing the transition that induced tumorigenesis is difficult. Single-cell analysis technologies, however, have emerged as powerful tools for identifying cell types or states, investigating cellular heterogeneity, and inferring transitions between cell states along developmental or differentiation trajectories in a high-dimensional gene expression space. In particular, pseudotime analysis enables the development of computational models of cell state transitions to predict system dynamics over time from static single-cell expression data^43^. These models are based on ergodicity, which is the assumption that a snapshot of a cell population at a single time point is equivalent to a trace pattern showing the evolution of a single cell over time. Therefore, applying pseudotime analysis to tumorigenesis with single-cell RNA sequencing data obtained from cancer and matched adjacent normal samples is a specific method for identifying cancer cell reversion based on an IC and EC. A pseudotime analysis of tumorigenesis can help predict the normal cell state and the transition state prior to tumor onset (Fig. 7). Furthermore, the availability of data of temporal transition gene expression associated with a cell state transition enables the inference of dynamic intracellular network models that can be used to identify cellular responses to extracellular inputs or network perturbations. These dynamic network models enable the exploration of the bistable properties of a transition state through an attractor landscape analysis. Once a transition state model has been constructed, (i) the dynamic network model of the cancer cell state can be established by introducing driver mutations, and (ii) optimal IC and EC candidates can be identified in complex networks by employing attractor-based control theories, such as the control kernel^51^ and stable motif control^52^. The suitability of candidate targets can be validated experimentally. Either existing therapeutics targeting these molecules can be evaluated, or the identified targets can be used for drug development to achieve therapeutic cancer reversion (Fig. 7).

### Case study: identifying IC and EC targets for cancer reversion by using single-cell RNA sequencing data from lung cancer samples

To show the usefulness of the proposed strategy for cancer reversion, we constructed a dynamic network model representing the transition state during tumorigenesis of lung cancer by using single-cell RNA-sequencing data (scRNA-seq) obtained from both tumor and adjacent normal tissues^62^ (Supplementary Fig. 1). A case study showing the use of this strategy illustrates the overall process, from obtaining scRNA-seq data to the identification of IC and EC targets of the dynamic network model (see Supplementary Text for details). To this end, we have limited our model to a small-scale network to illustrate the proposed control strategy, and an arbitrary kinetic parameter set was chosen to ensure a bistable cell state.

**Fig. 8:**
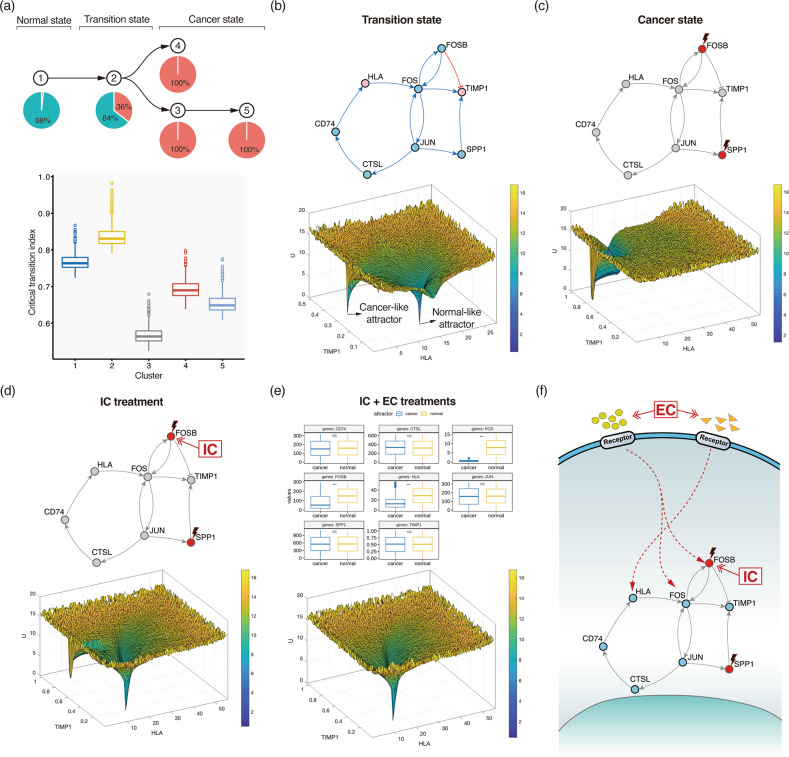
Identifying IC and EC targets for cancer reversion by using single-cell RNA-sequencing data obtained with lung cancer samples. **a** The subtrajectory from the normal cell cluster to the cancer cell clusters ran through the intermediate cluster (top) and the critical transition index of each cluster (bottom). Pie charts represent the composition of the normal and cancer cells in each cluster. **b** The gene regulatory network of the transition state (top) and the corresponding potential landscape (bottom). Output nodes are pink. HLA is a meta-gene representing genes with the same function in network topology, such as HLA-A, HLA-B, HLA-C, etc. **c** The rewired network in cells in the cancer state (top) and the corresponding potential landscapes (bottom). Genetic alterations to FOSB and SPP1 are shown in red. **d** Changes in the potential landscape after IC treatment. **e** Changes in the potential landscape after IC and EC treatments. Box plots showing comparisons of the two sets of initial states, one converges to a normal attractor and one that converges to a cancer-like attractor (NS: not statistically significant, ***p < 0.001). **f** A schematic showing the cancer reversion strategy with an IC that targets FOSB and an EC that targets certain receptors activating FOSB, FOS, and HLA by mediating signaling.

Cancer is initiated by the accumulation of genetic alterations. Therefore, we inferred a pseudotime-based order of tumorigenic steps based on single-nucleotide variants (SNVs) detected from scRNA-seq data by employing a computational method for use with a trajectory inference based on SNP information (TBSP) algorithm^63^ (Supplementary Fig. 2). We also employed the SCDIFF algorithm^64^, which has been suggested to be useful for analyzing cell differentiation trajectories on the basis of time-series scRNA-seq data, to obtain a subtrajectory of a normal cell population undergoing tumorigenesis and thus transitioning into a cancer cell population (Supplementary Fig. 3). The resulting subtrajectory contained a root cluster, where normal cells were dominant, end clusters with cancer cells, and an intermediate cluster where normal and cancer cells coexisted (Fig. 8a, top). Interestingly, the critical transition index, which is defined by gene‒gene correlation divided by cell‒cell correlation^44^, of the intermediate cluster was higher than that of the other clusters, suggesting that the intermediate cluster corresponded to the transition state caused by tumorigenesis (Fig. 8a, bottom). To construct a dynamic network model representing the transition state, differentially expressed genes (Supplementary Table 1) between cells in the transition state and cells in the cancer state were incorporated with prior knowledge of gene interactions obtained from STRING^65^, Omnipath^66^, and Human Signaling Network^67^ analyses (Supplementary Fig. 4). The final core network consisted of eight genes with a coupled feedback loop formed by FOSB/FOS/JUN, a subnetwork related to antigen, such as HLA, presentation and a subnetwork related to metastasis, which included factors such as TIMP1 (Fig. 8b, top). Estimation of the optimal kinetic parameters for a dynamic network model in systems biology is generally a challenge^68,69^. For this case study, we employed the sRACIPE^70^ algorithm to identify a kinetic parameter set that showed bistable cells, that is, a stable state comprising cells with a normal phenotype (low TIMP1 and high HLA levels) and cells with a cancer phenotype (high TIMP1 and low HLA levels) (Supplementary Table 2 and Supplementary Fig. 5).

Quantifying the attractor landscape is useful for understanding the bistable cells in the transition state. The potential landscape $U\left( {TIMP1,HLA} \right) = - \ln P(TIMP1,HLA)$^71^ of the transition state shows two distinct attractors: a normal-like attractor at low TIMP1 and high HLA levels and a cancer-like attractor at high TIMP1 and low HLA levels (Fig. 8b. bottom), where *P* represents the probability density of the cell states (see the Supplementary Text for details). Rewiring the regulatory network by altering genes in lung cancer, such as FOSB^72,73^ and SPP1^74,75^, changes the potential landscape; specifically, the normal-like attractor disappears and the cancer-like attractor shifts to a more malignant state (Supplementary Fig. 6a and Fig. 8c). The ultimate goal of using an IC is to drive this cancer state to a new transition state where normal- and cancer-like attractors coexist in the potential landscape. To find an IC target, we performed a perturbation simulation analysis for all the genes and found that upregulation of FOSB induced the reappearance of a normal-like attractor in the potential landscape (Fig. 8d). In addition, the ultimate goal of an EC is to block any state transition trajectory that leads to the cancer-like attractor while not inhibiting states converging only to a normal-like attractor. We traced all the initial states that converged to a cancer-like attractor and excluded the corresponding trajectories from the potential landscape (Supplementary Fig. 6b, c). The resulting landscape included only a normal-like attractor (Fig. 8e), suggesting that, via this system regulation, the cells behaved as normal cells under EC conditions. Comparing the two sets of initial states that converged to normal- and cancer-like attractors, we found that the levels of FOS, FOSB, and HLA were significantly decreased in the states with trajectories converging to the cancer-like attractor (Fig. 8e). This finding indicates that an EC, namely, drugs activating the upstream signaling pathways of FOS, FOSB, and HLA, can be implemented (Fig. 8f).

This case study shows the implementation of an IC and EC by constructing a gene regulatory network model and simulating its dynamic behaviors upon the potential landscape. Although this example illustrates the whole process proposed for inducing cancer reversion, from obtained single-cell data from a cancer patient to the identification of IC and EC targets, limitations are noted. In contrast to estimating optimal kinetic parameters from time-series data, we heuristically determined a specific parameter set that ensured cell bistability. Recent advances in single-cell transcriptomics have led to many dynamic network inference methods, such as SCODE for differential equation models and BTR and SCNS for Boolean network models (see Review articles^76,77^). However, due to the difficulty in identifying transitioning cells and their sparsity in the state space, modeling cell state transitions and controlling them are still outstanding challenges in biology and computational science. Another recent study proposed an approach for generating dynamic network models showing transitions between distinct states on the basis of omics data^78^. Applying these methods for understanding transitions to various types of omics data may enable us to estimate an optimal parameter set to fit real data and to better identify the optimal IC and EC targets for cancer reversion.

## Conclusion and future perspectives

A better understanding of the IC and EC targets contributing to cancer reversion will lead to new strategies for treating cancer and preventing tumorigenesis while reducing the risk of adverse effects on normal cells and preventing drug resistance. From a practical point of view, the proposed control strategy does not necessarily aim to restore cancer cells to their original state prior to tumorigenesis. In contrasts, finding traces of the original normal state of the normal cells from cancer patients and driving cancer cells to a state as close as possible to the original state is a more realistic approach to cancer reversion. An IC plays a role tracing normal cells, whereas an EC is critical for erasing any trace of cancer cells by allowing only a normal cell state in the epigenetic landscape. In our model framework, reverted cancer cells can be retransform into cancer cells when the EC treatment is halted because the cancer state exists in the landscape. Therefore, an EC treatment regimen may be required for a long time, even after cancer cells have been reverted to normal cells, to prevent cancer recurrence.

Cancers controlled or managed with continuous or repetitive treatments of EC drugs for long periods may be regarded as chronic diseases, similar to diabetes, asthma, and heart disease. Specifically, cancer reversion is not considered a cure, but it is “controlled” or “managed” because the signs and symptoms of cancer disappear after EC treatment. Although an EC treatment will likely not completely eliminate the chance of cancer recurrence, the ability to manage cancer as a chronic disease may help patients maintain a high quality of life throughout their cancer treatments. With this new paradigm, we may define a new “normal” in cancer therapy as “living with cancer risk,” a condition in which the risk of a cancer recurring is controlled effectively with an EC regimen. This model inspires future investigation into the development of additional and specific control strategies for cancer reversion, including methods to systematically identify IC and EC targets via multiomics data and to explore control targets with synergistic action for complete cancer reversion. Furthermore, the theoretical framework of our cancer reversion model based on attractor landscapes suggested in this review is not limited to cancer research and can be used to study other diseases induced by cell state transitions, including the EMT and cell reprogramming.

## Supplementary information

Supplementary Information

Supplementary Table S1

Supplementary Table S2

## References

[1] Kenny PA, Bissell MJ (2003). Tumor reversion: correction of malignant behavior by microenvironmental cues. Int. J. Cancer.

[2] Askanazy M (1907). Die Teratome nach ihrem Bau, ihrem Verlauf, ihrer Genese und im Vergleich zum experimentellen Teratoid. Verhandl. Dtsch. Pathol. Gesellsch..

[3] Cho K-H (2017). Cancer reversion, a renewed challenge in systems biology. Curr. Opin. Syst. Biol..

[4] Mintz B, Illmensee K (1975). Normal genetically mosaic mice produced from malignant teratocarcinoma cells. Proc. Natl Acad. Sci. USA.

[5] Lo-Coco F (2016). Targeted therapy alone for acute promyelocytic leukemia. N. Engl. J. Med..

[6] Cicconi L (2016). PML-RARalpha kinetics and impact of FLT3-ITD mutations in newly diagnosed acute promyelocytic leukaemia treated with ATRA and ATO or ATRA and chemotherapy. Leukemia.

[7] Burnett AK (2015). Arsenic trioxide and all-trans retinoic acid treatment for acute promyelocytic leukaemia in all risk groups (AML17): results of a randomised, controlled, phase 3 trial. Lancet Oncol..

[8] Lo-Coco F (2013). Retinoic acid and arsenic trioxide for acute promyelocytic leukemia. N. Engl. J. Med..

[9] Dow LE (2015). Apc restoration promotes cellular differentiation and reestablishes crypt homeostasis in colorectal cancer. Cell.

[10] Telerman A, Amson R (2009). The molecular programme of tumour reversion: the steps beyond malignant transformation. Nat. Rev. Cancer.

[11] Tuynder M (2002). Biological models and genes of tumor reversion: cellular reprogramming through tpt1/TCTP and SIAH-1. Proc. Natl Acad. Sci. USA.

[12] Martincorena I (2018). Somatic mutant clones colonize the human esophagus with age. Science.

[13] Martincorena I (2015). Tumor evolution. High burden and pervasive positive selection of somatic mutations in normal human skin. Science.

[14] Kaufman CK (2016). A zebrafish melanoma model reveals emergence of neural crest identity during melanoma initiation. Science.

[15] Lee S (2020). Network inference analysis identifies SETDB1 as a key regulator for reverting colorectal cancer cells into differentiated normal-like cells. Mol. Cancer Res..

[16] Brock A, Krause S, Ingber DE (2015). Control of cancer formation by intrinsic genetic noise and microenvironmental cues. Nat. Rev. Cancer.

[17] Powers S, Pollack RE (2016). Inducing stable reversion to achieve cancer control. Nat. Rev. Cancer.

[18] Vogel A, Pollack R (1973). Isolation and characterization of revertant cell lines. IV. Direct selection of serum-revertant sublines of SV40-transformed 3T3 mouse cells. J. Cell Physiol..

[19] Proietti S (2020). Tumor reversion and embryo morphogenetic factors. Semin. Cancer Biol..

[20] Bizzarri M, Giuliani A, Cucina A, Minini M (2020). Redifferentiation therapeutic strategies in cancer. Drug Discov. Today.

[21] Bizzarri M, Cucina A, Proietti S (2017). Tumor reversion: mesenchymal-epithelial transition as a critical step in managing the tumor-microenvironment cross-talk. Curr. Pharm. Des..

[22] Takahashi K, Yamanaka S (2006). Induction of pluripotent stem cells from mouse embryonic and adult fibroblast cultures by defined factors. Cell.

[23] Ohnishi K (2014). Premature termination of reprogramming in vivo leads to cancer development through altered epigenetic regulation. Cell.

[24] Mizrachi Y, Naranjo JR, Levi BZ, Pollard HB, Lelkes PI (1990). PC12 cells differentiate into chromaffin cell-like phenotype in coculture with adrenal medullary endothelial cells. Proc. Natl Acad. Sci. USA.

[25] Arnold JT, Lessey BA, Seppala M, Kaufman DG (2002). Effect of normal endometrial stroma on growth and differentiation in Ishikawa endometrial adenocarcinoma cells. Cancer Res..

[26] Mulas C, Chaigne A, Smith A, Chalut KJ (2021). Cell state transitions: definitions and challenges. Development.

[27] Huang, S. & Kauffman, S. A. in E*ncy*clopedia *o*f Com*plexity and Systems Science* (ed. Meyers, R. A.) 1180–1213 (Springer New York, 2009).

[28] Huang, S, Eichler G, Bar-Yam Y, Ingber DE (2005). Cell fates as high-dimensional attractor states of a complex gene regulatory network. Phys. Rev. Lett..

[29] Waddington, C. H. *The Strategy of the Genes; a Discussion of Some Aspects of Theoretical Biology* (Allen & Unwin, 1957).

[30] Huang S, Ernberg I, Kauffman S (2009). Cancer attractors: a systems view of tumors from a gene network dynamics and developmental perspective. Semin. Cell Dev. Biol..

[31] Choi M, Shi J, Zhu Y, Yang R, Cho K-H (2017). Network dynamics-based cancer panel stratification for systemic prediction of anticancer drug response. Nat. Commun..

[32] Choi SR, Hwang CY, Lee J, Cho KH (2022). Network analysis identifies regulators of basal-like breast cancer reprogramming and endocrine therapy vulnerability. Cancer Res..

[33] Kim Y, Choi S, Shin D, Cho KH (2017). Quantitative evaluation and reversion analysis of the attractor landscapes of an intracellular regulatory network for colorectal cancer. BMC Syst. Biol..

[34] Cho SH, Park SM, Lee HS, Lee HY, Cho KH (2016). Attractor landscape analysis of colorectal tumorigenesis and its reversion. BMC Syst. Biol..

[35] Cho KH, Joo JI, Shin D, Kim D, Park SM (2016). The reverse control of irreversible biological processes. WIRES Syst. Biol. Med..

[36] Choi M, Shi J, Jung SH, Chen X, Cho KH (2012). Attractor landscape analysis reveals feedback loops in the p53 network that control the cellular response to DNA damage. Sci. Signal..

[37] Shen-Orr SS, Milo R, Mangan S, Alon U (2002). Network motifs in the transcriptional regulation network of *Escherichia coli*. Nat. Genet..

[38] Alon U (2007). Network motifs: theory and experimental approaches. Nat. Rev. Genet..

[39] Thomas R, Thieffry D, Kaufman M (1995). Dynamical behaviour of biological regulatory networks—I. Biological role of feedback loops and practical use of the concept of the loop-characteristic state. Bull. Math. Biol..

[40] Kaufman M, Soule C, Thomas R (2007). A new necessary condition on interaction graphs for multistationarity. J. Theor. Biol..

[41] Kim JR, Yoon Y, Cho KH (2008). Coupled feedback loops form dynamic motifs of cellular networks. Biophys. J..

[42] Scheffer M (2012). Anticipating critical transitions. Science.

[43] Moris N, Pina C, Arias AM (2016). Transition states and cell fate decisions in epigenetic landscapes. Nat. Rev. Genet..

[44] Mojtahedi M (2016). Cell fate decision as high-dimensional critical state transition. PLoS Biol..

[45] Poovathingal SK, Kravchenko-Balasha N, Shin YS, Levine RD, Heath JR (2016). Critical points in tumorigenesis: a carcinogen-initiated phase transition analyzed via single-cell proteomics. Small.

[46] Martincorena I (2017). Universal patterns of selection in cancer and somatic tissues. Cell.

[47] Tomasetti C, Marchionni L, Nowak MA, Parmigiani G, Vogelstein B (2015). Only three driver gene mutations are required for the development of lung and colorectal cancers. Proc. Natl Acad. Sci. USA.

[48] Shin D, Lee J, Gong JR, Cho KH (2017). Percolation transition of cooperative mutational effects in colorectal tumorigenesis. Nat. Commun..

[49] Slotine, J.-J. E. & Li, W. *Applied Nonlinear Control*. Vol. 199 (Prentice hall Englewood Cliffs, NJ, 1991).

[50] Fiedler B, Mochizuki A, Kurosawa G, Saito D (2013). Dynamics and control at feedback vertex sets. I: Informative and determining nodes in regulatory networks. J. Dyn. Differ. Equ..

[51] Kim J, Park SM, Cho KH (2013). Discovery of a kernel for controlling biomolecular regulatory networks. Sci. Rep..

[52] Zanudo JG, Albert R (2015). Cell fate reprogramming by control of intracellular network dynamics. PLoS Comput. Biol..

[53] Pina C (2012). Inferring rules of lineage commitment in haematopoiesis. Nat. Cell Biol..

[54] Nahta R, O’Regan RM (2010). Evolving strategies for overcoming resistance to HER2-directed therapy: targeting the PI3K/Akt/mTOR pathway. Clin. Breast Cancer.

[55] Jaeger S (2017). Quantification of pathway cross-talk reveals novel synergistic drug combinations for breast cancer. Cancer Res..

[56] Klinger B (2013). Network quantification of EGFR signaling unveils potential for targeted combination therapy. Mol. Syst. Biol..

[57] Prahallad A (2012). Unresponsiveness of colon cancer to BRAF(V600E) inhibition through feedback activation of EGFR. Nature.

[58] Nguyen LK, Kholodenko BN (2016). Feedback regulation in cell signalling: lessons for cancer therapeutics. Semin. Cell Dev. Biol..

[59] Paull EO (2021). A modular master regulator landscape controls cancer transcriptional identity. Cell.

[60] Califano A, Alvarez MJ (2017). The recurrent architecture of tumour initiation, progression and drug sensitivity. Nat. Rev. Cancer.

[61] Bennett RL, Licht JD (2018). Targeting Epigenetics in Cancer. Annu. Rev. Pharmacol. Toxicol..

[62] Lambrechts D (2018). Phenotype molding of stromal cells in the lung tumor microenvironment. Nat. Med..

[63] Ding J, Lin C, Bar-Joseph Z (2019). Cell lineage inference from SNP and scRNA-Seq data. Nucleic Acids Res..

[64] Ding J (2018). Reconstructing differentiation networks and their regulation from time series single-cell expression data. Genome Res.

[65] Szklarczyk D (2021). The STRING database in 2021: customizable protein-protein networks, and functional characterization of user-uploaded gene/measurement sets. Nucleic Acids Res..

[66] Turei D, Korcsmaros T, Saez-Rodriguez J (2016). OmniPath: guidelines and gateway for literature-curated signaling pathway resources. Nat. Methods.

[67] Cui Q (2007). A map of human cancer signaling. Mol. Syst. Biol..

[68] Shin S-Y (2014). The switching role of β-adrenergic receptor signalling in cell survival or death decision of cardiomyocytes. Nat. Commun..

[69] Sreenath SN, Cho K-H, Wellstead P (2008). Modelling the dynamics of signalling pathways. Essays Biochem..

[70] Kohar V, Lu M (2018). Role of noise and parametric variation in the dynamics of gene regulatory circuits. NPJ Syst. Biol. Appl..

[71] Zhang X, Chong KH, Zhu L, Zheng J (2020). A Monte Carlo method for in silico modeling and visualization of Waddington’s epigenetic landscape with intermediate details. Biosystems.

[72] Daraselia N (2012). Molecular signature and pathway analysis of human primary squamous and adenocarcinoma lung cancers. Am. J. Cancer Res..

[73] Kim DS, Lee WK, Park JY (2020). Association of FOSB exon 4 unmethylation with poor prognosis in patients with late‑stage non‑small cell lung cancer. Oncol. Rep..

[74] Tang H, Chen J, Han X, Feng Y, Wang F (2021). Upregulation of SPP1 is a marker for poor lung cancer prognosis and contributes to cancer progression and cisplatin resistance. Front. Cell Dev. Biol..

[75] Yi X (2022). SPP1 facilitates cell migration and invasion by targeting COL11A1 in lung adenocarcinoma. Cancer Cell Int..

[76] Todorov H, Cannoodt R, Saelens W, Saeys Y (2019). Network inference from single-cell transcriptomic data. Methods Mol. Biol..

[77] Nguyen H, Tran D, Tran B, Pehlivan B, Nguyen T (2021). A comprehensive survey of regulatory network inference methods using single cell RNA sequencing data.. Brief. Bioinform..

[78] Rukhlenko OS (2022). Control of cell state transitions. Nature.

