# Supplementary material for: Critical transition and reversion of tumorigenesis
Source: Exp Mol Med. 2023 Apr 3;55(4):692–705. doi: 10.1038/s12276-023-00969-3 (PMC10167317; doi:10.1038/s12276-023-00969-3)
Supplement: Supplementary file 1 — Supplementary Information [file 12276_2023_969_MOESM1_ESM.pdf]

Critical Transition and Reversion of Tumorigenesis

Dongkwan Shin[1,2] and Kwang-Hyun Cho[1*]

[1]Department of Bio and Brain Engineering, Korea Advanced Institute of Science and Technology (KAIST), Daejeon, 34141, Republic of Korea

[2]Bioinformatics Branch, Division of Cancer Data Science, National Cancer Center, Goyang-si, Gyeonggi-do, 10408, Republic of Korea

Supplementary Information

Supplementary Text

Supplementary Fig. 1- 6

Supplementary Table 1- 2

[*]Corresponding author. E-mail: ckh@kaist.ac.kr, Phone: +82-42-350-4325, Fax:+82-42-350-4310, Web: http://sbie.kaist.ac.kr/

Supplementary Text

Case study: Identifying IC and EC targets for cancer reversion by using single cell RNA-sequencing data of lung cancer

To implement cancer reversion strategy using single cell data, we reconstructed a dynamic network model of lung cancer transition state as a case study by using single cell RNA-sequencing data (scRNA-seq) from tumor and adjacent normal tissues[1]. Developing detailed dynamic models of molecular regulatory networks is one of the primary challenges in systems biology as it requires sophisticated technique in determining both the regulatory structure of a network and kinetic parameters of each regulation from sufficient time-series data. Therefore, in this case study, we focused on a small-scale network model that is sufficient to conceptually demonstrate the proposed cancer reversion strategy based on IC and EC, and determined arbitrary kinetic parameter values so that the network model has bistability on its attractor landscape (see Supplementary Fig. 1 for entire workflow for the reconstruction of a dynamic network model). With this simple model we explored all the genes to find a key target gene that can change the attractor landscape into a desired shape, rather than using complex network control methods such as FVS, control kernel and stable motifs.

1. Inferring pseudo-time based on genetic alterations

Cancer is known to be developed by the accumulation of genetic alterations. Therefore, inferring a pseudo-time trajectory of tumorigenesis needs to be based on genetic alterations rather than gene expression. To infer pseudo-time ordering by genetic alterations, we employed a computational method for trajectory inference based on SNP information (TBSP)[2] that detects significant single nucleotide variations on RNA sequences from scRNA-seq data (Supplementary Fig. 2(a)). SNVs detected from scRNA-seq data could be sufficiently

informative to correctly cluster the cells and align the clusters along the development of cancer because they are expressed and their functions are readily linked to cancer phenotypes. We analyzed scRNA-seq data (221 normal cells and 883 tumor cells) on tumor (core, middle, and edge) and matched normal tissue from a lung cancer patient (patient 5 in the reference[1]). To identify informative SNVs from scRNA-seq data, we aligned the data using Cellranger (version 4.0) and we used GATK RNA-seq variant calling pipeline to call the vcfs from aligned bam files. After calling SNP at single cell levels, we used the TBSP algorithm to find out the order of tumorigenesis based on accumulation of DNA variation. From the result of the TBSP, we identified pseudo-time ordering of the development of lung cancer (Supplementary Fig. 2(b)). We found that some normal cells originating from tumor and normal tissue are located in very early time, T = 0, 1, and 2, and that there is an intermediate state where normal and cancer cells coexist (Supplementary Fig. 2(b) and (c)).

2. Reconstructing the tumorigenic trajectory from normal to cancer cell states

Although the intermediate state (T = 2 in Supplementary Fig. 2(c)) is likely a transition state, it is necessary to check whether the critical transition index, which is defined by gene-gene correlation divided by cell-cell correlation[3], is maximized in the transition state. We selected randomly 500 genes during 1000 trials and calculated the critical transition index in each pseudo-time by SNP, showing that values at T = 2 is lower than other time points (Supplementary Fig. 3(a)), which implicates that the cluster at T = 2 is not a transition state. It may originate from the heterogeneity in the cluster at each time point. Heterogeneity is a typical characteristic of cancer. So, even if the cells exist at the same SNP pseudo-time, they can show different profiles in terms of gene expression. It means that we cannot assume that cells at the next time point (T = t+1) necessarily evolve from the cells at the previous time point (T = t). Therefore, one must identify different cell populations within a specific time point by clustering

and to link them over time to find out a correct trajectory from normal to cancer states. To do this, we employed the SCDIFF algorithm[4] to obtain the sub-trajectory that explains the dynamic cell trajectory of tumorigenesis from the root cell cluster, where normal tissue-derived cells are dominant, to end clusters that contain only tumor tissue-derived cells through an intermediate cluster that includes mixture of both tissues (Supplementary Fig. 3(b)). Interestingly, the critical transition index of cluster 2 in the sub-trajectory is higher than the others, suggesting that the cluster 2 corresponds to a transition state (Supplementary Fig. 3(c)).

3. Reconstructing a molecular regulatory network of the transition state

To reconstruct a dynamic network model representing the transition state, we obtained 21 differentially expressed genes and a relevant transcript factor between the transition state (cluster 2) and a tumor cell state (cluster 3) (Supplementary table 1). In this case study, we used existing knowledge of molecular interaction network including STRING[5], Omnipath[6], and Human Signaling Network[7] to reconstruct a mechanism-based molecular regulatory network. First, we extracted the protein interaction network with 21 core genes from the STRING database, which includes both functional and physical associations and additional proteins connecting two input genes. After removing respiratory-related protein modules from the extracted network, the resulting network consisted of modules related to antigen processing and presentation, immune system, and cellular responses to stimuli (Supplementary Fig. 4(a)). Next, we determined the direction and regulatory sign of each link by using the Omnipath and Human Signaling Network databases, and excluded links that could not be determined or links that were incompatible between two databases. Genes that have the same function in network topology were merged, such as HLA representing HLA-A, HLA-B, HLA-C, HLA-DPA1, and so on. As a result, the final core network with eight genes consists of a coupled feedback loop formed of FOSB/FOS/JUN, a part related to antigen presentation such as HLA, and the other

part related to metastasis such as TIMP1 (Supplementary Fig. 4(b)).

4. Determining kinetic parameters of the molecular regulatory network of the transition state

The estimation of kinetic parameters for a dynamic network model is a critical challenge. In this case study, we used the sRACIPE[8] algorithm to determine kinetic parameters so that our dynamic network model exhibits a bistability, where one stable state is related to a phenotype of normal cells and the other is that of cancer cells.

Our network model can be described by the ordinary differential equations (ODEs), where the effect of each regulation is formulated as a Hill type function, as follows:

$$d\frac{y(1)}{dt} = \frac{G_1}{FC_{21}} * (FC_{21} + \frac{1 - FC_{21}}{1 + \left(\frac{y(2)}{TH_{21}}\right)^{N_{21}}}) - K_1 * y(1);$$

$$d\frac{y(2)}{dt} = \frac{G_2}{FC_{12}}\left(FC_{12} + \frac{1 - FC_{12}}{1 + \left(\frac{y(1)}{TH_{12}}\right)^{N_{12}}}\right)\frac{1}{FC_{32}}\left(FC_{32} + \frac{1 - FC_{32}}{1 + \left(\frac{y(3)}{TH_{32}}\right)^{N_{32}}}\right)\frac{1}{FC_{72}}\left(FC_{72} + \frac{1 - FC_{72}}{1 + \left(\frac{y(7)}{TH_{72}}\right)^{N_{72}}}\right) - K_2 * y(2);$$

$$d\frac{y(3)}{dt} = \frac{G_3}{FC_{23}} * (FC_{23} + \frac{1 - FC_{23}}{1 + \left(\frac{y(2)}{TH_{23}}\right)^{N_{23}}}) - K_3 * y(3);$$

$$d\frac{y(4)}{dt} = \frac{G_4}{FC_{34}} * (FC_{34} + \frac{1 - FC_{34}}{1 + \left(\frac{y(3)}{TH_{34}}\right)^{N_{34}}}) - K_4 * y(4);$$

$$d\frac{y(5)}{dt} = \frac{G_5}{FC_{35}} * (FC_{35} + \frac{1 - FC_{35}}{1 + \left(\frac{y(3)}{TH_{35}}\right)^{N_{35}}}) - K_5 * y(5);$$

$$d\frac{y(6)}{dt} = \frac{G_6}{FC_{56}} * (FC_{56} + \frac{1 - FC_{56}}{1 + \left(\frac{y(5)}{TH_{56}}\right)^{N_{56}}}) - K_6 * y(6);$$

$$d\frac{y(7)}{dt} = \frac{G_7}{FC_{67}} * (FC_{67} + \frac{1 - FC_{67}}{1 + \left(\frac{y(6)}{TH_{67}}\right)^{N_{67}}}) - K_7 * y(7);$$

$$\frac{y(8)}{dt} = G_8\left(FC_{18} + \frac{1 - FC_{18}}{1 + \left(\frac{y(1)}{TH_{18}}\right)^{N_{18}}}\right)\frac{1}{FC_{28}}\left(FC_{28} + \frac{1 - FC_{28}}{1 + \left(\frac{y(2)}{TH_{28}}\right)^{N_{28}}}\right)\frac{1}{FC_{48}}\left(FC_{48} + \frac{1 - FC_{48}}{1 + \left(\frac{y(4)}{TH_{48}}\right)^{N_{48}}}\right) - K_8 * y(8);$$

where $y = (FOSB, FOS, JUN, SPP1, CTSL, CD74, HLA, TIMP1)$ is the expression vector of all the genes, G_i and K_i are the maximum production rate and the degradation rate of the gene i, and FC_{ji}, N_{ji}, and TH_{ji} are the maximum fold change of the gene i, Hill coefficient of regulation, and the threshold level for the regulatory link from gene j to gene i.

Next, we generated 6,000 models by randomizing each parameter within the range, simulated the dynamics of each model with a particular set of parameters, and repeated the simulations for 10,000 times with randomly selected initial conditions. From the ensemble of models, we can analyze the robust dynamical properties of the input network topology. Our network model shows two stable steady states in 2D probability density map of the simulated gene expression data projected to the first two principal components (Supplementary Fig. 5(a)). From the hierarchical clustering analysis, we observe that there are two main clusters, cluster 1 (up-regulated TIMP1 and SPP1 and down-regulated HLA) and cluster 2 (the opposite pattern to cluster 1), as shown in Supplementary Fig. 5(b). Considering that HLA is an integrated gene related to antigen presentation and TIMP1 plays an important role in promoting tumorigenesis and metastasis, cluster 1 corresponds to a cancer-like state whereas cluster 2 is close to a normal-like state. For a bistable gene regulatory network, we determined a specific set of

kinetic parameters (Supplementary table 2) with which the dynamics of the model converges to two stable steady states, (high TIMP1, low HLA) and (low TIMP1, high HLA).

5. Quantifying attractor landscape of the transition state

Quantifying attractor landscape of the transition state by the dynamic network model is useful for understanding the bistability of the transition state and the corresponding phenotypic characteristic of each stable state. To do this, we employed a method based on Monte Carlo simulation which estimates the probability distribution, P, of cellular states by collecting a large number of time-course simulations with random initial conditions[9]. By projecting all the trajectories into a 2-dimensional plane of TIMP1 and HLA, which represent metastasis and antigen presentation respectively, we can obtain the quasi-potential $U(TIMP1, HLA) = -\ln P(TIMP1, HLA)$ of the transition state, as shown in Supplementary Fig. 6(a), where the potential landscape shows two distinct attractors; normal (low TIMP1 and high HLA)- and cancer (high TIMP1 and low HLA)-like attractors.

Next, we investigated how the shapes of the attractors in the potential landscape are changed when cancer genes in lung cancer rewire the gene regulatory network. FOSB was significantly down-regulated in both LUAD and LUSC, indicating that it may have a tumor suppressor function in the progression of lung cancer[10,11]. The expression of SPP1 in lung cancer tissues was significantly higher than in normal tissues, and patients with high SPP1 expression were also correlated with poor clinical prognosis[12,13]. Our perturbation analysis based on these evidences showed that the down-regulation of FOSB by increasing the degradation rate by 16 times induced the disappearance of the normal-like attractor and the appearance of a long valley between the cancer-like attractor and some more malignant state. In addition, when the SPP1 level was up-regulated by decreasing the degradation rate by 16 times, the position and the shape of the normal-like attractor was almost unchanged, but the cancer-like attractor was

shifted toward the high level of TIMP1, that is, more malignant state. Finally, we combined both perturbations to obtain a more malignant cancer state which has only a more malignant cancer-like attractor (Fig. 8(c) and Supplementary Fig. 6(a)).

6. IC and EC for cancer reversion

The ultimate goal of IC is to send the cancer state to a new transition state where normal- and cancer-like attractors coexist in the potential landscape. To find such a target, we executed perturbation simulation for all the genes and found that the up-regulation of FOSB by increasing the production rate induced the reappearance of a normal-like attractor on the potential landscape, although its shape is not the same as that of the original transition state (Fig. 8(d) and Supplementary Fig. 6(a)). The ultimate goal of EC is to block any trajectory converging to the cancer-like attractor and thus to allow only initial states that converge to the normal-like attractor. We traced all the initial states converging to the cancer-like attractor and excluded those trajectories in the potential landscape. The resulting landscape confirmed that there is only a normal-like attractor (Fig. 8(e) and Supplementary Fig. (b)), suggesting that this cellular system can behave as a normal cell under the EC condition. Comparing the two set of initial states that converge to the normal- and cancer-like attractor respectively, we found that the levels of FOS, FOSB, and HLA are significantly decreased in initial states converging to the cancer-like attractor (Fig. 8(e) and Supplementary Fig. 6(c)). In other words, EC can be implemented by drugs that activate the upstream signaling pathways of FOS, FOSB, and HLA (Fig. 8(f)).

This case study demonstrated the implementation of IC and EC by constructing a gene regulatory network model and simulating its dynamic behaviors on the potential landscape. Although this example showed the whole process for cancer reversion from single cell data to

the identification of IC and EC targets, it has a crucial limitation. Without estimating parameters from the single cell transcriptomic data, an arbitrary parameter set was chosen while ensuring bistability. Recent advances in single cell transcriptomics have fostered the development of the dynamic network inference methods based on differential equations, such as SCODE, and Boolean model, such as BTR and SCNS (see Review articles[14,15]). However, due to the difficulty in identifying transition cells and their sparsity in a state space, modeling cell state transitions and controlling them is still an outstanding challenge in biology and computational science. A recent great work proposed an approach for modeling dynamic network models during transitions between distinct cell states by using omics data[16]. Applying such methods for understanding transitions to various types of omics data such as single cell data will enable to estimate a unique parameter set to fit the real data and to further identify more exact IC and EC targets for cancer reversion.

References

1 Lambrechts, D. *et al.* Phenotype molding of stromal cells in the lung tumor microenvironment. *Nat. Med.* **24**, 1277-1289 (2018). https://doi.org:10.1038/s41591-018-0096-5

2 Ding, J., Lin, C. & Bar-Joseph, Z. Cell lineage inference from SNP and scRNA-Seq data. *Nucleic Acids Res.* **47**, e56 (2019). https://doi.org:10.1093/nar/gkz146

3 Mojtahedi, M. *et al.* Cell Fate Decision as High-Dimensional Critical State Transition. *PLoS Biol.* **14**, e2000640 (2016). https://doi.org:10.1371/journal.pbio.2000640

4 Ding, J. *et al.* Reconstructing differentiation networks and their regulation from time series single-cell expression data. *Genome Res.* **28**, 383-395 (2018). https://doi.org:10.1101/gr.225979.117

5 Szklarczyk, D. *et al.* The STRING database in 2021: customizable protein-protein networks, and functional characterization of user-uploaded gene/measurement sets. *Nucleic Acids Res.* **49**, D605-D612 (2021). https://doi.org:10.1093/nar/gkaa1074

6 Turei, D., Korcsmaros, T. & Saez-Rodriguez, J. OmniPath: guidelines and gateway for literature-curated signaling pathway resources. *Nat. Methods* **13**, 966-967 (2016). https://doi.org:10.1038/nmeth.4077

7 Cui, Q. *et al.* A map of human cancer signaling. *Mol. Syst. Biol.* **3**, 152 (2007). https://doi.org:10.1038/msb4100200

8 Kohar, V. & Lu, M. Role of noise and parametric variation in the dynamics of gene regulatory circuits. *NPJ Syst. Biol. Appl.* **4**, 40 (2018). https://doi.org:10.1038/s41540-018-0076-x

9 Zhang, X., Chong, K. H., Zhu, L. & Zheng, J. A Monte Carlo method for in silico modeling and visualization of Waddington's epigenetic landscape with intermediate details. *Biosystems* **198**, 104275 (2020). https://doi.org:10.1016/j.biosystems.2020.104275

10 Daraselia, N. *et al.* Molecular signature and pathway analysis of human primary squamous and adenocarcinoma lung cancers. *Am. J. Cancer Res.* **2**, 93-103 (2012).

11 Kim, D. S., Lee, W. K. & Park, J. Y. Association of FOSB exon 4 unmethylation with poor prognosis in patients with late-stage non-small cell lung cancer. *Oncol. Rep.* **43**, 655-661 (2020). https://doi.org:10.3892/or.2019.7431

12 Tang, H., Chen, J., Han, X., Feng, Y. & Wang, F. Upregulation of SPP1 Is a Marker for Poor Lung Cancer Prognosis and Contributes to Cancer Progression and Cisplatin Resistance. *Front. Cell Dev. Biol.* **9**, 646390 (2021). https://doi.org:10.3389/fcell.2021.646390

13 Yi, X. *et al.* SPP1 facilitates cell migration and invasion by targeting COL11A1 in lung adenocarcinoma. *Cancer Cell Int.* **22**, 324 (2022). https://doi.org:10.1186/s12935-022-02749-x

14 Todorov, H., Cannoodt, R., Saelens, W. & Saeys, Y. Network Inference from Single-Cell Transcriptomic Data. *Methods Mol. Biol.* **1883**, 235-249 (2019). https://doi.org:10.1007/978-1-4939-8882-2_10

15 Nguyen, H., Tran, D., Tran, B., Pehlivan, B. & Nguyen, T. A comprehensive survey of regulatory network inference methods using single cell RNA sequencing data. *Brief. Bioinform.* **22** (2021). https://doi.org:10.1093/bib/bbaa190

16 Rukhlenko, O. S. *et al.* Control of cell state transitions. *Nature* **609**, 975-985

(2022). https://doi.org:10.1038/s41586-022-05194-y

Supplementary Figures

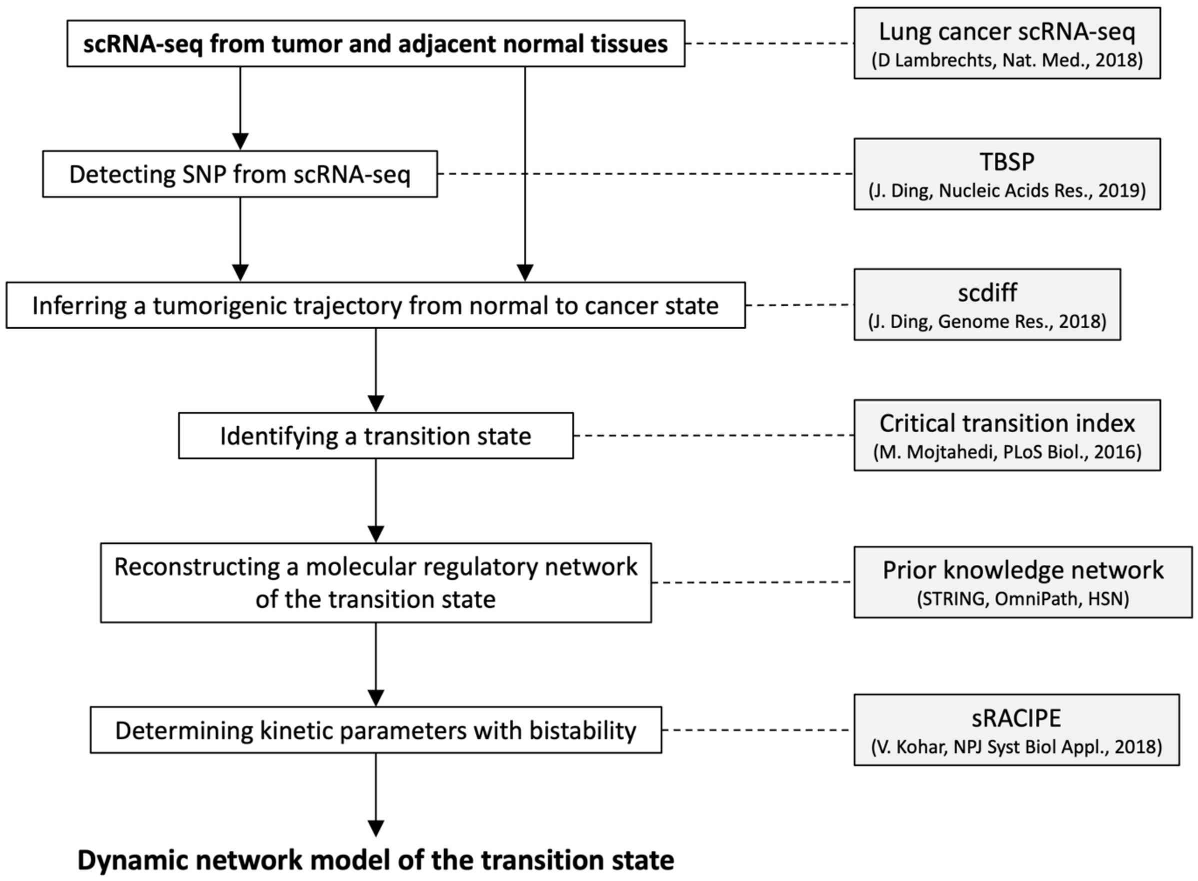

Supplementary Fig. 1. Workflow for reconstructing a dynamic network model representing the transition state by using single cell RNA-sequencing data from lung cancer.

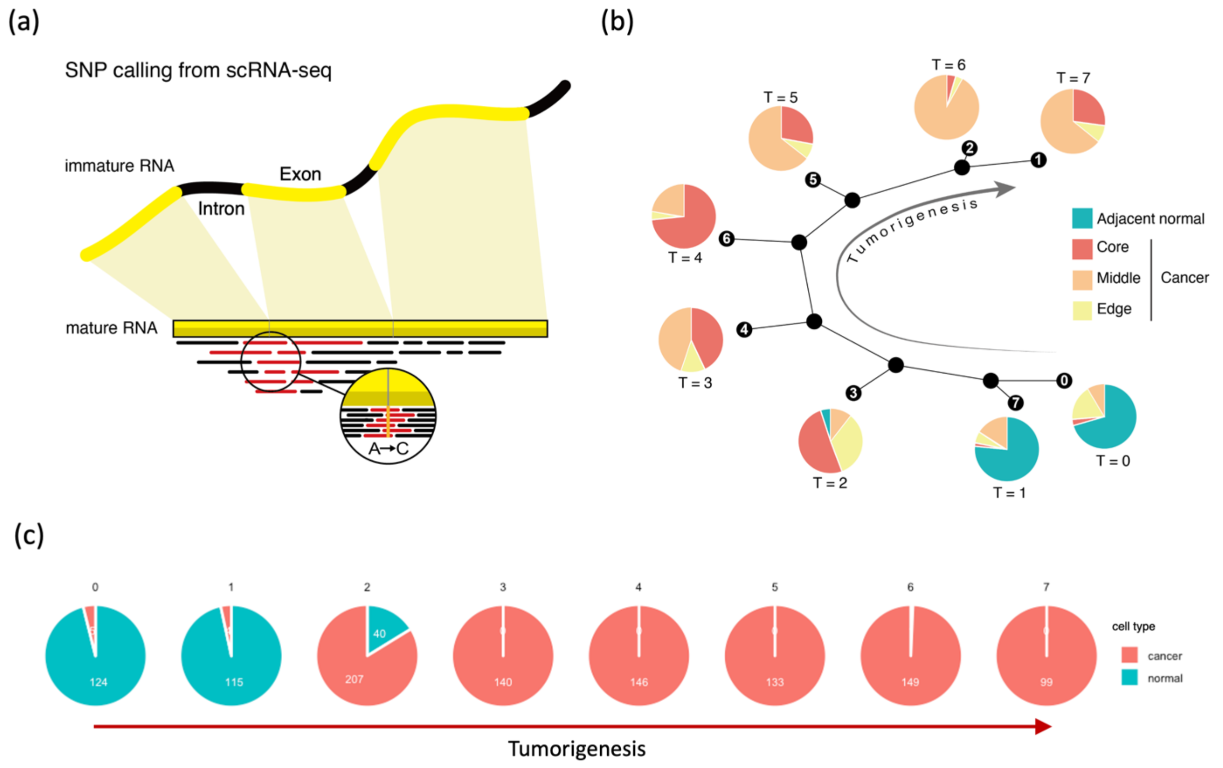

Supplementary Fig. 2. Inference of pseudo-time based on SNVs detected from scRNA-seq. (a) Schematic of SNP calling from scRNA-seq. Introns are spliced out of the immature mRNA to form mature mRNA where only the coding exons remain. The TBSP algorithm detects SNVs different from the reference genome and identifies informative SNPs to correctly cluster the cells along the pseudo-time axis. (b) Arrangement of clusters along the pseudo-time axis. Pie chart shows the composition of cell origin of each cluster. (c) Pie chart of the composition of normal and cancer cells of each cluster.

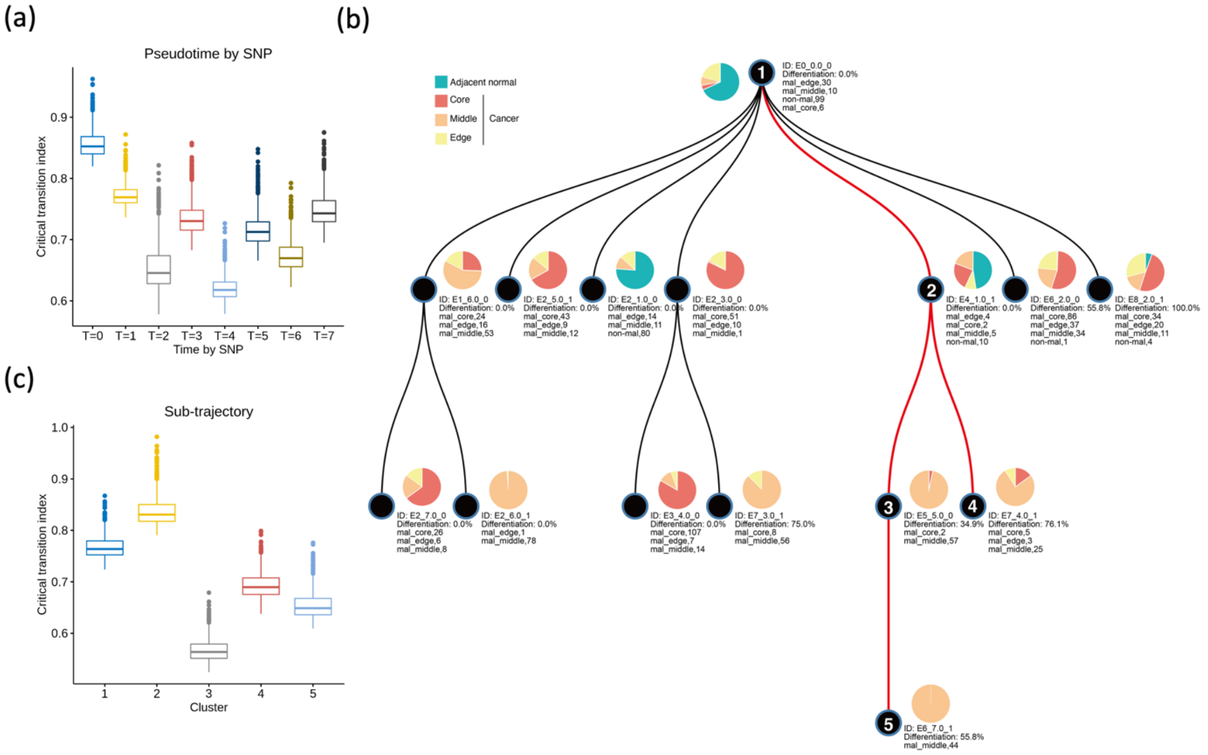

Supplementary Fig. 3. Reconstructing tumorigenic sub-trajectory from normal to cancer cell states. (a) Critical transition index of each cluster along pseudo-time based on variants. (b) Inferred tumorigenic trajectory by integrating expression similarity with the pseudo-time based on variants. The sub-trajectory from the normal cell cluster to the cancer cell clusters through the intermediate cluster is shown in red. (c) The critical transition index for each cluster in the sub-trajectory.

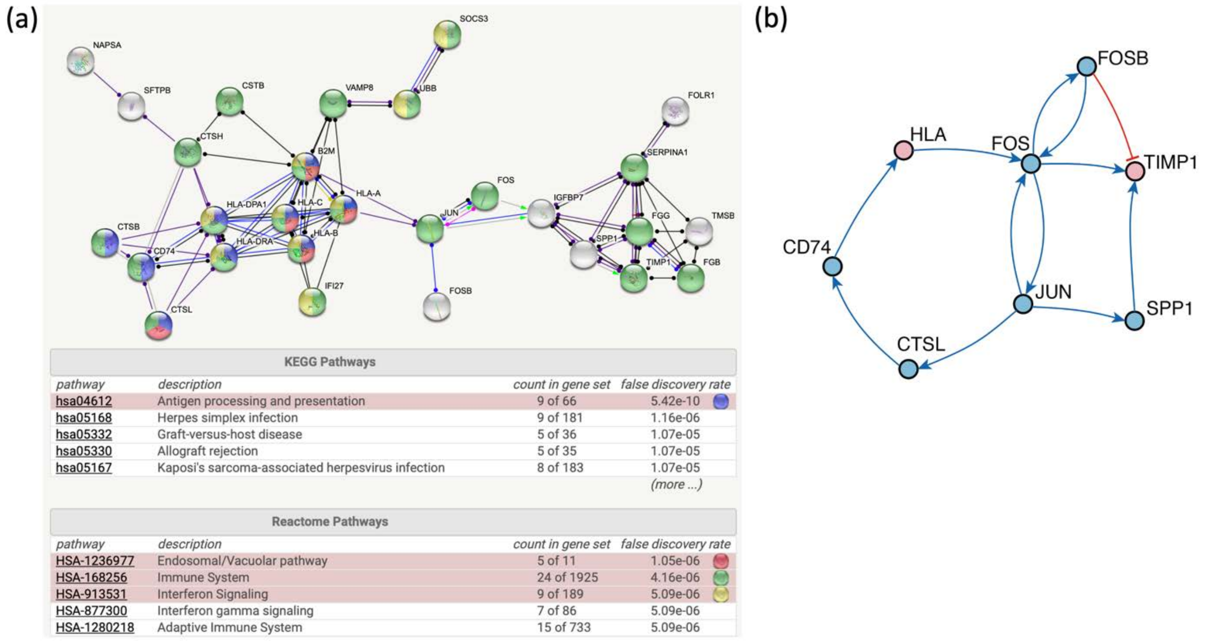

Supplementary Fig. 4. Reconstructing a molecular regulatory network of the transition state. (a) The protein interaction network extracted from the STRING database. The network consists of genes relevant to antigen processing and presentation (KEGG pathways), immune system (Reactome pathways), and cellular responses to stimuli (Biological process of the Gene Ontology, data not shown). (b) Resulting molecular regulatory network with directed and signed links. The output nodes are shown in pink.

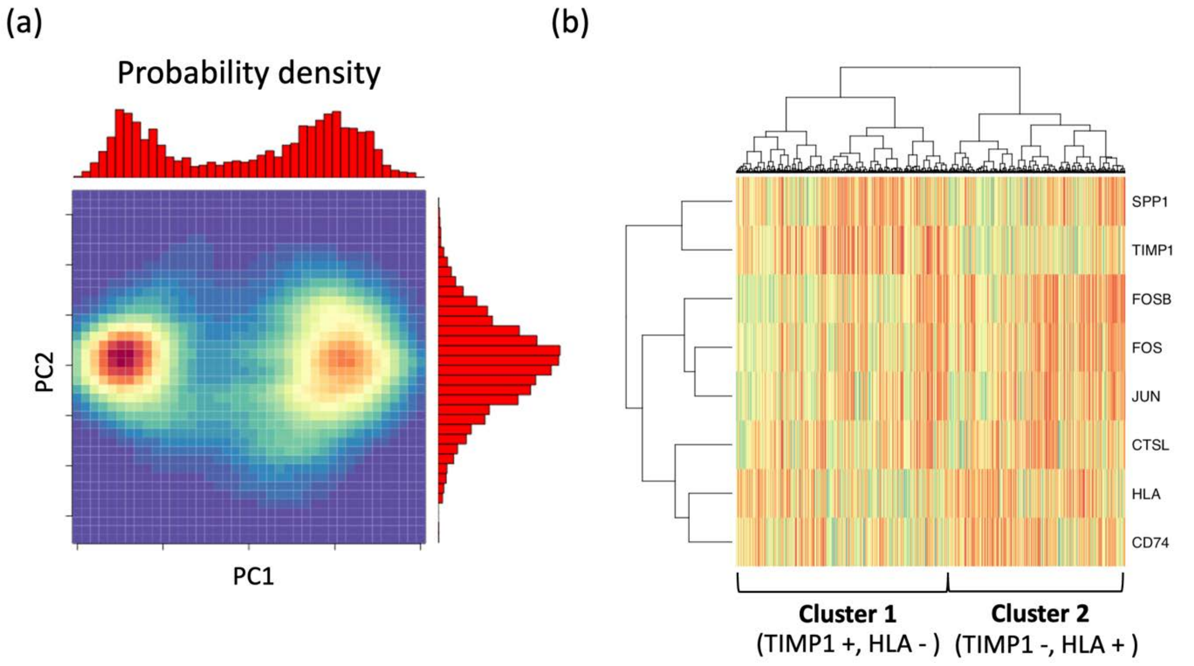

Supplementary Fig. 5. Determining kinetic parameters of the network model. (a) Probability density map of the sRACIPE-generated expression data projected onto the PCA space. (b) Hierarchical clustering analysis of simulated gene expression with randomly selected parameters. Each column corresponds to a stable steady state generated from the algorithm.

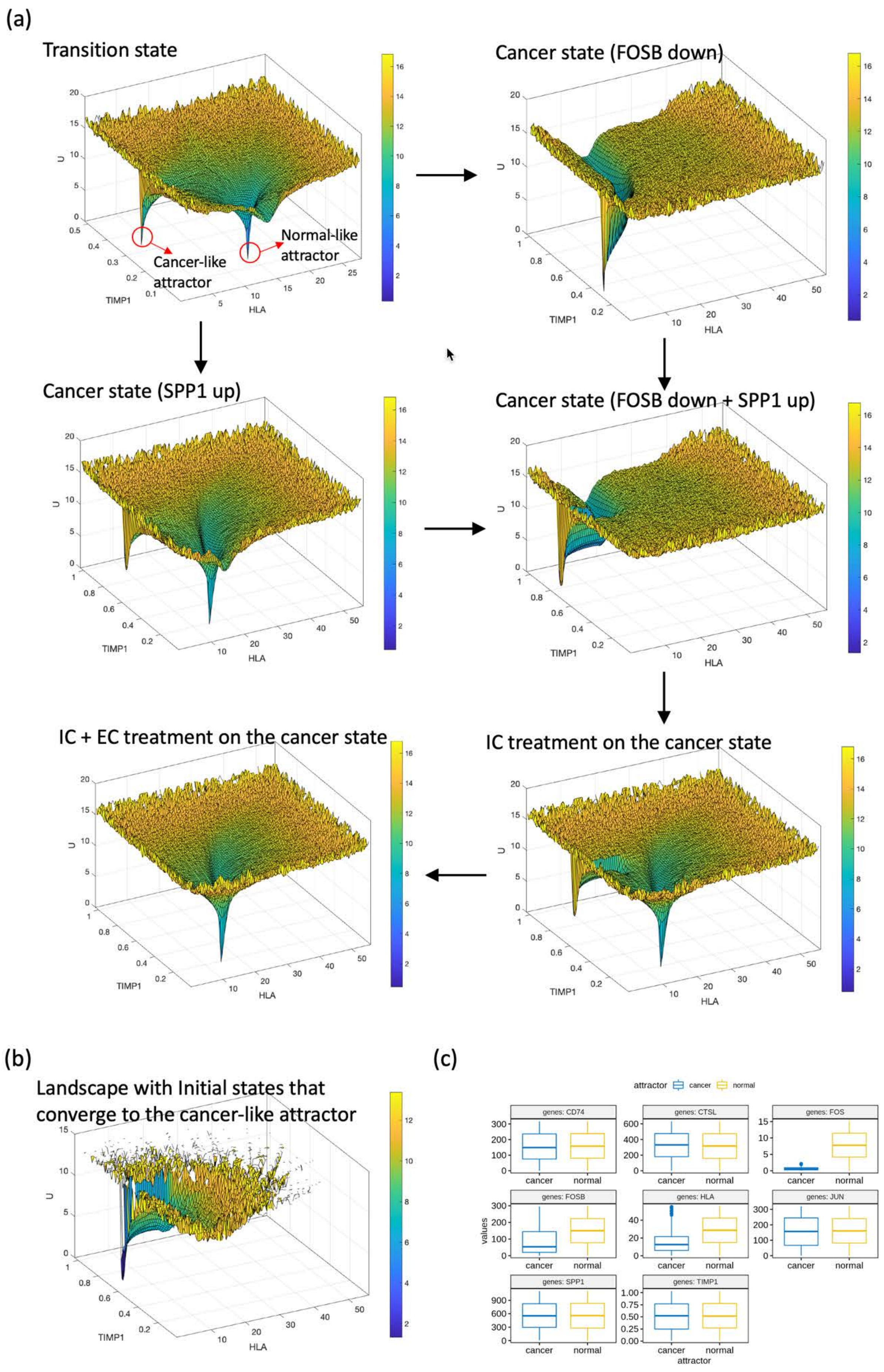

(a)
Transition state
Cancer-like attractor
Normal-like attractor
Cancer state (FOSB down)
Cancer state (SPP1 up)
Cancer state (FOSB down + SPP1 up)
IC + EC treatment on the cancer state
IC treatment on the cancer state
U
TIMP1
HLA
(b)
Landscape with Initial states that converge to the cancer-like attractor
(c)
attractor cancer normal
genes: CD74
genes: CTSL
genes: FOS
genes: FOSB
genes: HLA
genes: JUN
genes: SPP1
genes: TIMP1
values
cancer
normal
attractor

Supplementary Fig. 6. Potential landscape of the transition state and its changes for IC and EC treatments. (a) Potential landscape of the transition state, the cancer state, a new transition state after the IC treatment, and a normal-like state after the IC and EC treatment. (b) Potential landscape with only with initial states that converge to the cancer-like attractor. (c) Comparison of the two set of initial states that converge to the normal- and cancer-like attractor respectively.

Supplementary Tables

Supplementary Table 1. The DEGs and TF list between the transition state (cluster 2) and the cancer state (cluster 3).

Supplementary Table 2. Kinetic parameters of the dynamic network model with bistability
